# Hypergolic Copper Cluster‐Based Covalent Organic Frameworks

**DOI:** 10.1002/advs.76306

**Published:** 2026-06-26

**Authors:** Yu‐Zhou Qiao, Cai Li, Wen‐Yang Jiao, Guoqiang Sun, Xiao‐Fei Liu, Shuang‐Quan Zang

**Affiliations:** ^1^ Key Laboratory of Special Functional Molecular Materials (Zhengzhou University) Ministry of Education College of Chemistry Zhengzhou University Zhengzhou China; ^2^ Henan Key Laboratory of Crystalline Molecular Functional Materials College of Chemistry Zhengzhou University Zhengzhou China

**Keywords:** carborane, covalent organic framework, hypergolic materials, trinuclear copper

## Abstract

Rational design of hypergolic materials that integrate rapid ignition with high energy density is of great significance for advancing propulsion technologies. Herein, we achieve synergistic enhancement in hypergolic performance by incorporating catalytic centers and energy units into a single covalent organic framework (COF) constructed from metal‐cluster nodes and carborane building blocks. A trinuclear copper cluster serves simultaneously as a structural building unit and a catalytic center, directing the formation of the reticular framework while activating an otherwise inert carborane‐based linker. The resulting COF inherits the hypergolic character of the copper clusters, exhibiting an ignition delay time of 40 ms with high‐test peroxide as the oxidizer, while the carborane cages impart a high energy density of 25.9 kJ g^−1^. Theoretical simulations confirm strong interfacial interactions and a low reaction energy barrier between COF and oxidizer, supporting the design concept of synergistic performance. This work establishes a rational design strategy for the precise construction of functionalized hypergolic framework materials.

## Graphical abstract

Hpergolic cluster‐based covalent organic framework (Cu_3_‐CB‐COF), assembled from aldehyde‐functionalized carborane monomers and trinuclear copper clusters, demonstrates a strong synergy between its catalytic copper nodes and energetic carborane backbones.

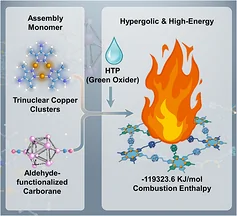

## 1 Introduction

Hypergolic materials, which ignite spontaneously upon contact with an oxidizer, are vital to aerospace and defense systems because of their rapid energy release and controllability [1, 2, 3, 4]. In hybrid rocket engines, replacing conventional inert fuels such as paraffin or hydroxyl‐terminated polybutadiene with hypergolic alternatives simplifies engine design by eliminating ignition delay and suppressing combustion instability, thereby improving overall reliability [5]. Current hypergolic fuels rely heavily on hydrazine derivatives, which despite high specific impulse pose serious safety and environmental concerns due to toxicity, low volumetric energy density, and hazardous handling [6, 7, 8]. The development of new hypergolic materials that combine environmental compatibility, low sensitivity, high energy density, and excellent thermal stability therefore remains an important challenge.

Boron‐rich compounds are promising candidates for high‐energy propellants, offering high volumetric energy density and combustion enthalpy [9, 10]. Recent research has focused on borohydride‐based ionic liquids as hypergolic fuels [11, 12, 13, 14]. Among them, carborane stand out for their high boron content and exceptional thermal and hydrolytic stability, which originate from their 3D cage structures and super aromatic character [15, 16]. Their chemical inertness, however, prevents spontaneous ignition, precluding direct use as hypergolic fuels. Previously, we addressed this using metal nanoclusters protected by carborane ligands, where the metal core acts as a catalytic hotspot to initiate combustion with oxidizers [17]. Yet such clusters—typically containing more than ten metal atoms—involve an inherent trade‐off: higher metal nuclearity lowers the energetic ligand‐to‐metal ratio and thus the theoretical energy density, while the dense ligand shell creates steric hindrance that shields the metal sites, hinders oxidizer diffusion, and suppresses electrophilic attack critical to the catalytic process.

Trinuclear copper clusters exhibit a planar, nine‐membered ring geometry stabilized by metal–metal interactions [18, 19]. Recent studies have shown that the three metal centers in such clusters can act cooperatively to activate small molecules such as O_2_ and H_2_O_2_ by stabilizing key reaction intermediates and reducing activation barriers—leading to their broad utility in catalysis [20, 21]. Compared with larger clusters, trinuclear copper species offer highly exposed, cyclic tri‐metal sites that display superior reactivity toward oxidizers [22]. Moreover, the higher proportion of energetic ligands in trinuclear complexes favors improved hypergolic performance. We therefore propose that trinuclear copper clusters can serve as efficient catalytic centers to trigger carborane ignition and promote energy release. Notably, these cyclic trinuclear complexes can also act as secondary building units for constructing framework materials thereby enabling diverse applications [23, 24, 25, 26].

Here we present a hypergolic cluster‐based covalent organic framework (COF), denoted Cu_3_‐CB‐COF, constructed from trinuclear copper clusters (Cu_3_‐NH_2_) as nodes and aldehyde‐functionalized carborane (CB‐CHO) as organic linkers (Figure 1). Functionally, the trinuclear copper clusters act as catalytic centers that successfully ignite the carborane components and enable substantial energy release. Upon contact with high‐test peroxide (HTP), Cu_3_‐CB‐COF shows a short ignition delay (ID) time of 40 ms. It also delivers a high calculated combustion enthalpy of ‐119323.6 kJ mol^−1^ and achieves a mass‐based energy density of 25.9 kJ g^−1^, attributable to the high carborane content within the framework. Theoretical calculations corroborate the catalytic function of the trinuclear copper active sites and help explain the superior performance of Cu_3_‐CB‐COF. The design and assembly of framework materials based on trinuclear copper clusters and carborane linkers offers a novel strategy for developing advanced hypergolic materials.

**FIGURE 1:**
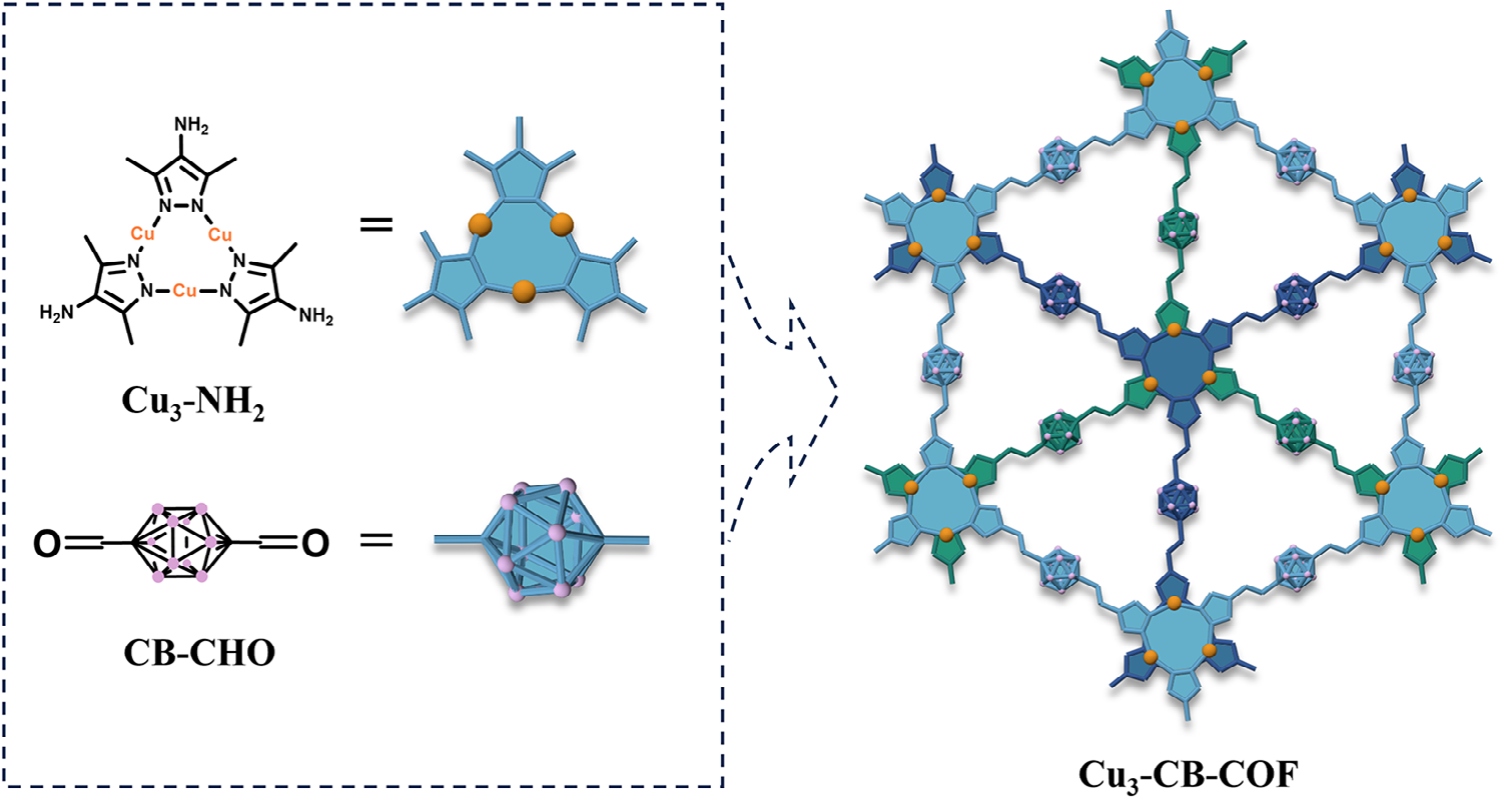
Schematic illustration of the synthesis of Cu_3_‐CB‐COF.

## 2 Results and Discussion

Cu_3_‐CB‐COF was synthesized via Schiff‐base condensation between Cu_3_‐NH_2_ and CB‐CHO. The CB‐CHO linker was prepared from p‐carborane and confirmed by ^1^H, ^13^C and ^11^B NMR spectroscopy (Figures S1–S3) [27]. The Cu_3_‐NH_2_ cluster was similarly verified by powder X‐ray diffraction (PXRD) and scanning electron microscope (Figures S4 and S5) [28]. The crystallinity and long‐range order of the assembled framework were assessed by PXRD [29]. PXRD of Cu_3_‐CB‐COF displays a strong peak at 2*θ* = 5.45° corresponding to the (110) plane, with additional reflections at 9.19°, 10.71°, and 26.13° (Figure 2a). Pawley refinement confirms an ABC stacking model in space group R3̅ with *a* = *b* = 32.7679 Å, *c* = 10.3581 Å (*R*_p_ = 4.69%, *R*_wp_ = 5.03%), consistent with the designed topology. The formation of imine linkages was verified by Fourier‐transform infrared (FT‐IR) spectra, solid‐state ^13^C cross‐polarization magic‐angle spinning (CP/MAS) NMR, and X‐ray photoelectron spectroscopy (XPS) [27, 30]. FT‐IR spectra show disappearance of N─H and ─CHO stretches and emergence of a C═N stretch, confirming Schiff‐base condensation (Figure 2b). Notably, the B─H stretching band of the carborane moiety remains unchanged, indicating the structural integrity of the carborane cages within the framework. The ^13^C CP/MAS NMR spectrum shows signals at 148 ppm (C═N) and 12 ppm (methyl groups), evidencing imine formation and node stability (Figure 2c). XPS analysis supports these findings: B 1s peaks at 189.3 eV (B─C) and 189.7 eV (B─B/B─H), and N 1s signals at 400.3, 399.3, and 398.1 eV, consistent with nitrogen environments (Figure S6).

**FIGURE 2:**
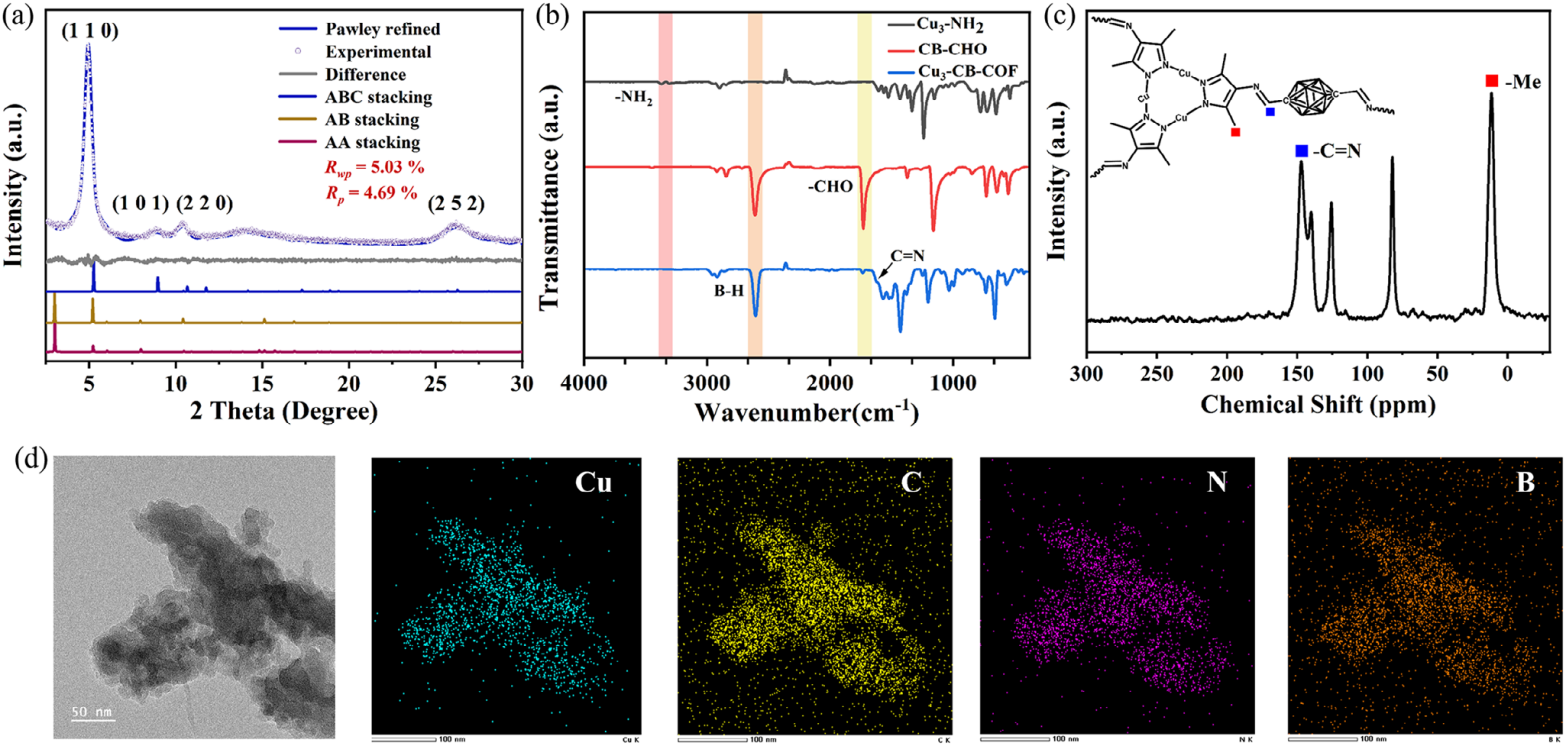
(a) PXRD patterns of Cu_3_‐CB‐COF. (b) FT‐IR spectra of Cu_3_‐NH_2_, CB‐CHO and Cu_3_‐CB‐COF. (c) ^13^C CP/MAS NMR spectrum of Cu_3_‐CB‐COF. (d) TEM image and elemental mapping images of Cu_3_‐CB‐COF.

X‐ray absorption fine structure (XAFS) measurements confirmed the structural integrity of the active Cu_3_‐NH_2_ within the material [31]. The absorption edge of Cu_3_‐CB‐COF indicates a Cu oxidation state close to +1, and no significant edge shift is observed relative to Cu_3_‐NH_2_, suggesting that the assembly of the COF framework does not alter the valence or structure of the trinuclear copper center (Figure S7). Furthermore, the peak at ≈1.5 Å in the Fourier‐transformed *R*‐space spectrum confirms the Cu─N coordination and the precise construction of active sites in Cu_3_‐CB‐COF (Figure S8). The porosity and pore structure of the assembled material were examined by N_2_ adsorption–desorption measurements at 77 K [32]. The Brunauer‐Emmett‐Teller (BET) surface area of Cu_3_‐CB‐COF was determined to be 421.1 m^2^ g^−1^. The isotherm shows a sharp uptake at low *P*/*P*_0_ (<0.05), indicating micropore filling, followed by multilayer adsorption. The profile combines Type I and Type IV features, suggesting coexisting micropores and mesopores. Pore size distribution reveals micropores at 0.51 and 1.88 nm and mesopores at 3.86 nm (Figures S9 and S10). Moreover, transmission electron microscopy (TEM) and energy‐dispersive X‐ray spectroscopy elemental mapping confirm the homogeneous distribution of Cu, C, N, and B throughout the material (Figure 2d).

The ID time, a key parameter for evaluating hypergolic performance, is defined as the interval between the first contact of the fuel with the oxidizer and the onset of ignition [33]. The hypergolic performance of samples with HTP was evaluated using a standard oxidizer‑fuel droplet test. In this test, a single droplet of HTP (30 *µL*) was added to a 5 mL tube containing 15 mg of the sample, and the ID time was recorded using a high‐speed camera at 1000 fps. Using HTP as the oxidizer, the unassembled Cu_3_‐NH_2_ precursor showed an ID time of 60 ms (Figure 3), confirming its role as a reactive node that imparts hypergolicity. Its combustion, however, produced only scattered, weak sparks, reflecting a lack of energetic components. By contrast, carborane‐integrated Cu_3_‐CB‐COF exhibited a shorter ID time of 40 ms and generated a bright reddish‐green flame roughly 7 cm in height (Figure 3). The reduced ID time is ascribed to the distinctive reducing ability of carborane, which accelerates the redox reaction with H_2_O_2_. This marked difference in combustion behavior aligns with the design concept: in the Cu_3_‐CB‐COF, the cyclic trinuclear copper clusters act as catalytic centers that activate the otherwise inert carborane, while the carborane themselves serve as high‐energy components that substantially boost energy release, leading to a far more vigorous combustion reaction. In addition, to evaluate the hypergolic potential of Cu_3_‐CB‐COF in composite propellants, a paraffin wax grain was drilled in the center, filled with Cu_3_‐CB‐COF (10 mg), and subjected to an ignition test using HTP as the oxidizer (Figure S11). The composite grain underwent a hypergolic reaction 70 ms after contact with HTP, producing a bright and intense flame, accompanied by the melting and combustion of the paraffin wax. This result demonstrates that Cu_3_‐CB‐COF can effectively trigger the ignition and combustion of paraffin wax fuel, showing clear potential as a reactive additive for hybrid rocket fuels.

**FIGURE 3:**
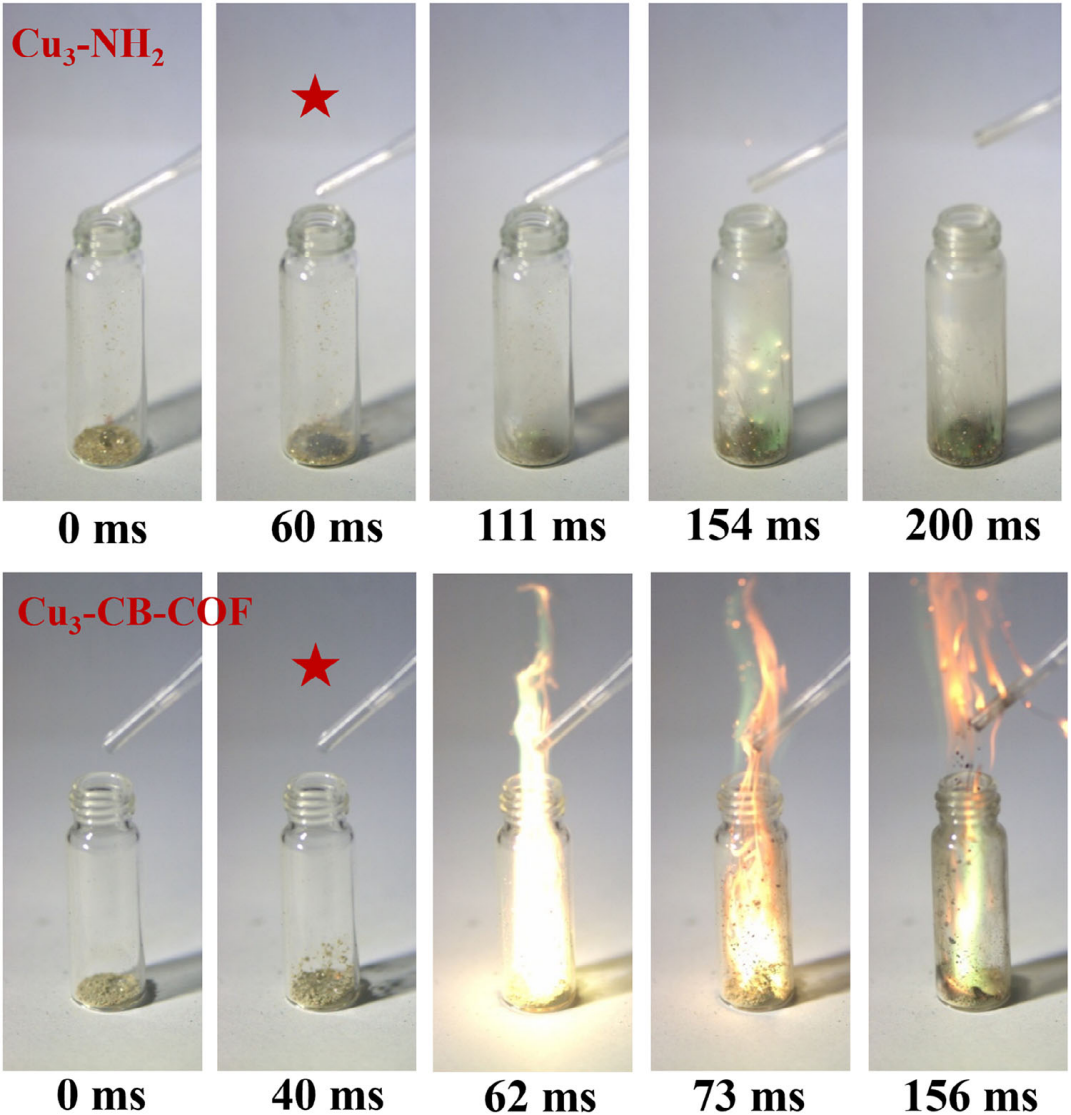
Hypergolicity drop tests of Cu_3_‐NH_2_ and Cu_3_‐CB‐COF with HTP.

Thermogravimetric analysis under nitrogen demonstrated the high thermal stability of Cu_3_‐CB‐COF (Figure S12). For solid hypergolic fuels, high mass energy density and combustion enthalpy increase propulsive energy, extending payload capacity and operational range. The standard molar enthalpies (Δ_c_ *H*) were determined to be ‐7669.5 kJ mol^−1^ for Cu_3_‐NH_2_ and ‐119323.6 kJ mol^−1^ for Cu_3_‐CB‐COF, corresponding to mass energy densities of 14.7 and 25.9 kJ g^−1^, respectively. For Cu_3_‐CB‐COF, the maximum specific impulse (*I*_sp_) reaches 250.5 s at an optimal oxidizer‐to‐fuel (O/F) mass ratio of 4, while Cu_3_‐NH_2_ achieves an *I*_sp_ of 235.5 s at an O/F ratio of 3 (Figure S13). Compared with previously reported hypergolic materials—including metal‐organic frameworks, high nuclearity clusters, and discrete molecules, Cu_3_‐CB‐COF delivers superior energetic performance (Table 1), offering a comparable or higher *I*_sp_ along with a more negative combustion enthalpy and a higher mass energy density [36, 37, 38, 39, 40, 41, 42]. These results indicate that, upon complete combustion, Cu_3_‐CB‐COF releases more energy per unit mass, leading to a more vigorous exothermic process and potentially greater thrust. The high mass energy density also facilitates storage and transport by reducing the fuel mass required for a given energy output. To confirm that these advantages originate from the carborane units rather than the COF architecture alone, we prepared a control material, Cu_3_‐Ph‐COF, by replacing the CB‐CHO linker with terephthalaldehyde (Figure S14). Cu_3_‐Ph‐COF was systematically characterized by XAFS, PXRD, ^13^C CP/MAS NMR, BET and TEM (Figures S7,S8, and S15–S19). Except for a comparable *I*_sp_ of 246.8 s at an O/F of 3 to that of Cu_3_‐CB‐COF (Figure S13), Cu_3_‐Ph‐COF exhibits a longer ID time (67 ms, Figure S20) and markedly lower energetic metrics than Cu_3_‐CB‐COF (Table 1), confirming carborane ligands enhance energy output. Together, these findings highlight the outstanding energy release characteristics of Cu_3_‐CB‐COF and support its potential as a solid hypergolic fuel.

**TABLE 1:** Hypergolic parameters of Cu_3_‐CB‐COF, Cu_3_‐Ph‐COF, Cu_3_‐NH_2_ and the reported energetic materials.

Fuel	ID (ms)		Δ_c_ *H* (kJ mol^−1^) ^a^	*E*_g_ (kJ g^−1^)	*I*_sp_ ^b^
	HTP	WFNA			
Cu_3_‐CB‐COF	40	216	−119323.6	25.9	250.5
Cu_3_‐Ph‐COF	67	—	−79866.3	19.9	246.8
Cu_3_‐NH_2_	60	—	−7669.5	14.7	235.5
Zn(AIm)_2_‐CB [34]	—	4	−6916.7	24.6	—
Cu_14B‐S_ [35]	3	—	−57925.6	24.4	217.7
CBA‐CuAg [17]	—	15	−77355.0	—	—
ZZU‐363 [5]	—	26	−46893.9	15.3	263.1
[Cu(DCA)2(1‐VIM)2]n [36]	—	28	—	27.9	208.0
Cu(PTe)_2_(CTrB)_2_ [37]	—	53	−10696.9	21.7	227.5
[Cu(MTZ)2(CTB)2]n [38]	—	30	−10083.0	22.6	247.0
Cu‐CTB‐VIm [39]	—	40	−11241.1	24.0	234.0
Zn(VIm)_2_ [40]	—	29	−4789.9	19.0	—
BmimDCA [41]	—	44	−6069.0	—	244.2
UDMH [42]	—	4.8	−1980.1	—	239.0

^a^ Calculated combustion enthalpy.

^b^ Specific impulse.

For hypergolic materials, assessing their stability is of critical importance for practical applications and safe handling. We evaluated the water and air stability of the three materials. PXRD results show that Cu_3_‐CB‐COF, Cu_3_‐Ph‐COF and Cu_3_‐NH_2_ retain their original crystalline phases after 14 d in water and 30 d under ambient, proving favorable structure stability (Figure S21). Furthermore, the contact angles of Cu_3_‐CB‐COF, Cu_3_‐Ph‐COF and Cu_3_‐NH_2_ were measured to be 97.01°, 86.52°, and 71.92°, respectively (Figure S22). The relatively higher contact angle yet shortest ID time of Cu_3_‐CB‐COF can be rationalized by the fact that the kinetic factors of the intrinsic chemical reaction dominate the ignition process, rather than the wettability of the macroscopic external surface.

To elucidate the structure–activity relationships underlying the hypergolic performance, we performed a series of theoretical calculations on Cu_3_‐NH_2_ and Cu_3_‐CB‐COF. Charge‐density difference analysis visualizes electron redistribution upon H_2_O_2_ adsorption. When H_2_O_2_ binds to the Cu sites, electron density transfers from the metal to the oxidizer, as seen in the distinct charge redistribution at the catalytic Cu centers (Figure S23)—a pattern consistent with strong interfacial interactions that facilitate O─O bond activation. Bader charge analysis quantifies this transfer: H_2_O_2_ adsorbed on Cu_3_‐CB‐COF gains a net charge of 0.1170 e^−^, larger than that on Cu_3_‐NH_2_ (Figure 4a). This indicates that embedding carborane ligands within the framework not only preserves but may even enhance interfacial electron transfer. Furthermore, we computed the reaction pathways and energy barriers for the redox process. In both systems, after initial adsorption (IS), H_2_O_2_ undergoes O─O cleavage at the metal core to form hydroxyl radicals (∙OH). The associated transition state (TS) energy barrier is lower for Cu_3_‐CB‐COF than for Cu_3_‐NH_2_, and the final state (FS) is more stable (Figure 4b and Figure S24), pointing to more favorable reaction kinetics and enhanced thermodynamic stability of the intermediates and products.

**FIGURE 4:**
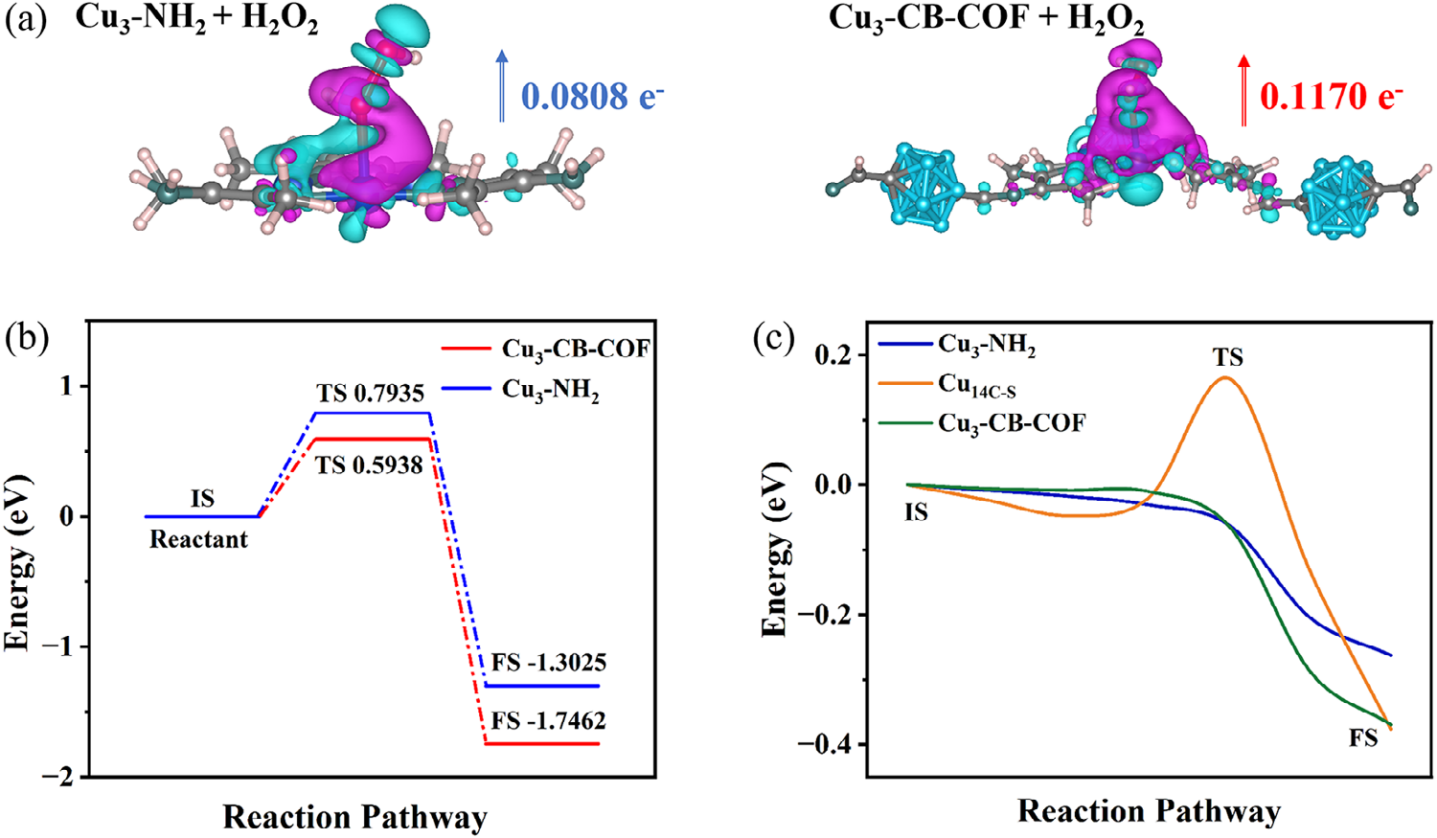
(a) Differential charge density maps of Cu_3_‐NH_2_ + H_2_O_2_ and Cu_3_‐CB‐COF + H_2_O_2_. (b) Free energy barrier diagram of hypergolic materials reacting with H_2_O_2_. (c) Diffusion barrier diagram for H_2_O_2_ diffusing to hypergolic materials.

The accessibility of catalytic active sites is critically influenced by steric constraints [29, 43]. To assess the advantage of the highly exposed metal sites in the trinuclear copper system, we performed comparative calculations using the larger‐nuclearity cluster Cu_14C‐S_ (ID = 6870 ms) [35], which contains carborane ligands but adopts a sterically crowded 3D structure. The diffusion barriers for H_2_O_2_ approaching the adsorption sites were evaluated for Cu_3_‐NH_2_, Cu_3_‐CB‐COF and Cu_14C‐S_. For both Cu_3_‐NH_2_ and Cu_3_‐CB‐COF, no discernible barrier (i.e., no transition state) was found along the diffusion coordinate; the potential energy decreases monotonically as H_2_O_2_ approaches the surface (Figure 4c and Figure S25). This indicates that the fully exposed metal sites provide a barrier‐free pathway, enabling the system to evolve directly toward a more stable configuration. In contrast, the metal core of Cu_14C‐S_ is densely shielded by surrounding ligands, resulting in pronounced steric hindrance. Consequently, H_2_O_2_ must overcome a substantial activation barrier to reach the Cu sites, hindering mass transport, limiting oxidant delivery to the catalytic centers, and ultimately restricting the overall reaction rate through a diffusion‐controlled process. These computational results support two key conclusions: first, assembling trinuclear copper secondary building units with carborane linkers into a framework enhances energetic performance while retaining catalytic reactivity; second, the structurally open metal sites in trinuclear copper units serve as effective catalytic centers for activating carborane‐based fuels.

## 3 Conclusions

Here, we report the first carborane‐based COF that functions as a high‐performance hypergolic fuel. The framework incorporates highly exposed trinuclear copper clusters, which remain unaggregated and provide readily accessible catalytic sites. Through reticular assembly with energetic carborane linkers, a synergistic proximity between catalytic centers and the fuel backbone is achieved, accelerating heat propagation during combustion and enabling rapid framework breakdown, intense energy release, and a short ID time. Supported by experimental and theory evidence, these results establish COFs as a promising platform for next‐generation hypergolic fuels and offer clear design principles for future energetic materials.

## Conflicts of Interest

The authors declare no conflicts of interest.

## Supporting information




**Supporting File 1**: advs76306‐sup‐0001‐SuppMat.docx.

**Supporting File 2**: advs76306‐sup‐0002‐VideoS1.mp4.

**Supporting File 3**: advs76306‐sup‐0003‐VideoS2.mp4.

**Supporting File 4**: advs76306‐sup‐0004‐VideoS3.mp4.

**Supporting File 5**: advs76306‐sup‐0005‐VideoS4.mp4.

## Data Availability

The data that support the findings of this study are available in the Supporting Information of this article.
