# Supplementary material for: Hypergolic Copper Cluster‐Based Covalent Organic Frameworks
Source: Adv Sci (Weinh). 2026 Jun 26:e76306. Online ahead of print. doi: 10.1002/advs.76306 (PMC13336892; doi:10.1002/advs.76306)
Supplement: Supplementary file 1 — Supporting File 1: advs76306‐sup‐0001‐SuppMat.docx. [file ADVS-9999-e76306-s003.docx]

**Supporting Information**

**Hypergolic Copper Cluster-Based Covalent Organic Frameworks**

*Yu-Zhou Qiao*^1^*, Cai Li*^1^, *Wen-Yang Jiao*^1^*, Guoqiang Sun*^*1^*, Xiao-Fei Liu*^*1^ *and Shuang-Quan Zang*^*1,2^

^1^ Key Laboratory of Special Functional Molecular Materials (Zhengzhou University), Ministry of Education, College of Chemistry, Zhengzhou University, Zhengzhou 450001, China.
^2^ Henan Key Laboratory of Crystalline Molecular Functional Materials, College of Chemistry, Zhengzhou University, Zhengzhou 450001, China.

E-mail: sungq@zzu.edu.cn; liuxiaofei@zzu.edu.cn; zangsqzg@zzu.edu.cn

**Section S1 Materials and Measurements**

**Materials and Reagents**

All chemicals and solvents obtained from suppliers were used without further purification.

**Measurements**

^1^H NMR spectra (400 MHz) were acquired on a Bruker AVANCE III HD 400 spectrometer using CDCl_3_ (δ = 7.26) as the deuterated solvent. ^13^C NMR spectra (100 MHz) were recorded on a Bruker Avance 400 spectrometer with CDCl_3_ (δ = 77) as the solvent. Powder X ray diffraction (PXRD) data were collected on a Rigaku D/Max 2500PC diffractometer equipped with a Cu Kα source (λ = 1.54178 Å). Samples were deposited on silicon substrates and scanned from 2θ = 2° to 30° with a step size of 0.01°. Fourier transform infrared attenuated total reflection (FT-IR ATR) spectra were recorded on a Bruker ALPHA spectrometer in the range 400-4000 cm^-1^. N_2_ adsorption-desorption isotherms were measured at 77 K on a Micromeritics ASAP 2460 analyzer after degassing the powder samples at 120 °C for 12 h. The specific surface area was calculated using the Brunauer-Emmett-Teller (BET) method, and pore size distributions were derived from non local density functional theory (NL DFT) models. Solid state ^13^C cross polarization magic angle spinning (CP/MAS) NMR spectra were obtained on a Bruker Avance Neo 400WB spectrometer. Scanning electron microscopy (SEM) images, energy dispersive X ray spectroscopy (EDS) and elemental mapping were performed on ZEISS-Sigma 300. High resolution transmission electron microscopy (HRTEM) was carried out on a JEOL JEM F200 microscope. X-ray absorption fine structure (XAFS) was measured at Anhui Absorption Spectroscopy Analysis Instrument Co., Ltd. (RapidXAFS 2M). X-ray absorption near-edge structure (XANES) spectra were processed with the Athena program. Thermogravimetric analysis (TGA) and differential scanning calorimetry (DSC) were conducted on a PerkinElmer STA 8000 thermal analyzer from room temperature to 600 °C at a heating rate of 10 °C min^-1^ under a continuous flow of high purity N_2_ (99.99%). Combustion enthalpies were determined using a high pressure oxygen bomb calorimeter (IDEA Science BCA 500). Typically, 0.1 g of sample was combusted in a crucible under 30 bar of O_2_, with benzoic acid pellets used as the reference standard.

**Combustion Calorimetry**

The change in internal energy of the materials was measured using oxygen bomb calorimetry under constant volume conditions. The constant-volume combustion heat (Δ_c_*U*) of Cu_3_-CB-COF, Cu_3_-Ph-COF and Cu_3_-NH_2_ are -119067.0018 kJ mol^-1^, -79810.5778 kJ mol^-1^ and -7662.0755 kJ mol^-1^, respectively. The constant-pressure combustion enthalpy (Δ_c_*H*) was calculated using the equation: Δ$\text{c}\text{H}\text{ = }\text{Δ}\text{c}\text{U}\text{ + }\text{Δ}\text{nRT}$, where Δ*n* is the change of the molar amount of gases during the reaction process, R = 8.314 J mol^-1^ K^-1^ and T = 298.15 K.

The combustion reaction equations are as follows:

Cu_3_-CB-COF: Cu_18_C_126_N_54_B_90_H_216_(s) + 256.5 O_2_(g) = 18 CuO(s) + 126 CO_2_(g) + 27 N_2_(g) + 45 B_2_O_3_(s) + 108 H_2_O(l)

Cu_3_-Ph-COF: Cu_18_C_162_N_54_H_162_(s) + 211.5 O_2_(g) = 18 CuO(s) + 162 CO_2_(g) + 27 N_2_(g) + 81 H_2_O(l)

Cu_3_-NH_2_: Cu_3_C_15_N_9_H_24_(s) + 22.5 O_2_(g) = 3 CuO(s) + 15 CO_2_(g) + 4.5 N_2_(g) + 12 H_2_O(l)

The calculated Δ_c_*H* values of Cu_3_-CB-COF, Cu_3_-Ph-COF and Cu_3_-NH_2_ are -119323.6 kJ mol^-1^, -79866.3 kJ mol^-1^ and -7669.5 kJ mol^-1^, respectively. The calculated *E*_g_ values of Cu_3_-CB-COF, Cu_3_-Ph-COF and Cu_3_-NH_2_ are 25.9 kJ g^-1^, 19.9 kJ g^-1^ and 14.4 kJ g^-1^, respectively.

Heat of formation was calculated by *Hess’s* law using below equation:

$$\text{∆}\text{f}\text{H}\text{θ}\text{m}\text{ }\text{= ∑∆}\text{f}\text{H}\text{θ}\text{m}\text{ (product) - ∆}\text{c}\text{H}\text{θ}\text{m}$$

Based on the known enthalpies of formation of products (Δ_f_*H*^θ^_m_ (CuO, s) = -157.3 kJ mol^-1^; Δ_f_*H*^θ^_m_ (CO_2_, g) = -393.51 kJ mol^-1^; Δ_f_*H*^θ^_m_ (B_2_O_3_, s) = -1273.5 kJ mol^-1^; Δ_f_*H*^θ^_m_ (H_2_O, l) = -285.8 kJ mol^-1^),^1^ the calculated Δ_f_*H* of Cu_3_-CB-COF, Cu_3_-Ph-COF and Cu_3_-NH_2_ are -21263.4 kJ·mol^-1^, -9862.7 kJ·mol^-1^ and -2134.6 kJ·mol^-1^, respectively.

**Sensitivity test**

The friction sensitivity (FS) was determined using a Bundesanstalt für Materialprüfung (BAM) friction tester. The impact sensitivity (IS) was measured on a BAM Fall Hammer Impact Sensitivity Tester (model BFH 12, OZM Research).

The sensitivity test results indicated that the Cu_3_‑CB‑COF is sensitive to external stimuli, including impact and friction (impact sensitivity: > 20 J, friction sensitivity: > 168 N).

**Calculation of specific impulse (*I*_sp_)**

The specific impulse of the composites was calculated using the Chemical Equilibrium with Applications (CEA) codes developed by NASA researchers ^2^. According to the formula *I*_sp_ = *k*$\sqrt{\text{T}\text{c}\text{/M}}$, the *I*_sp_ depends on the gaseous combustion temperature (*T_c_*) and the average molecular mass of the combustion gas (M). Additionally, Isp is influenced by physical parameters such as oxidizer-to-fuel ratio (*O/F*), chamber pressure (*P_c_*), ambient pressure (*P_e_*), ambient temperature, and nozzle expansion ratio. In this work, the ambient temperature was set to 298.15 K, with *P_c_/P_e_* = 70 atm, *O/F* = 1.0-8.0 and equilibrium flow conditions assumed during expansion.

**Section S2 Synthesis of Cu_3_-NH_2_, CB-CHO, Cu_3_-CB-COF and Cu_3_-Ph-COF**

**Synthesis of Cu_3_-NH_2_**

Cu_3_-NH_2_ was synthesized following a reported procedure with slight modifications.^3^ Briefly, Cu_2_O (43 mg, 0.30 mmol) and 3,5-dimethyl-1H-pyrazol-4-amine (100.0 mg, 0.90 mmol) were dissolved in a mixture of ethanol (4 mL) and pyridine (0.4 mL) in a 10 mL Pyrex tube. The tube was degassed via three freeze-pump-thaw cycles and sealed under flame. After ultrasonication to ensure dispersion, the mixture was heated at 120 °C for 72 h. The yellow needle crystals were collected, washed with ethanol and dried under vacuum for 6 h, yielding 55.0 mg (64 %).

**Synthesis of CB-CHO**

CB‑CHO was synthesized via a two‑step lithiation‑formylation sequence.^4^ In a nitrogen‑filled glovebox, p‑carborane (5.9 mmol, 850 mg) was dissolved in anhydrous THF (50 mL) in a 100 mL Schlenk flask equipped with a magnetic stir bar and cooled to -78 °C. A solution of n‑BuLi in hexanes (2.5 M, 13.5 mmol, 5.4 mL) was added dropwise over 10 min. The reaction mixture was stirred at -78 °C for 30 min, then gradually warmed to room temperature over 45 min. After recooling to -40 °C, methyl formate (30 mmol, 1.85 mL) was added slowly. The mixture was allowed to warm to room temperature and stirred for 12 h. The reaction was quenched with 2 M HCl (3 mL). The THF was removed under reduced pressure, and the resulting aqueous mixture was extracted with hexane (3 × 20 mL). The combined organic extracts were concentrated in vacuo. The crude solid was purified by flash column chromatography on silica gel (eluent: hexane/dichloromethane, 1:2, v/v). After removal of solvents, the product was dried at 60 °C under vacuum for 12 h to afford CB‑CHO as a white crystalline powder (430 mg, 35 % yield).

**Synthesis of Cu_3_-CB-COF**

Cu_3_-CB‑COF was assembled via solvothermal imine condensation. A mixture of Cu_3_‑NH_2_ (0.02 mmol, 10.50 mg) and CB‑CHO (0.03 mmol, 6.00 mg) in o‑dichlorobenzene/n‑butanol/6 M acetic acid (5:5:1 v/v, 1.1 mL) was placed in a 10 mL Pyrex tube. The tube was degassed through three freeze-pump-thaw cycles and sealed under vacuum. It was then heated at 120 °C for 72 h. The resulting green crystalline solid was collected by filtration, washed repeatedly with anhydrous tetrahydrofuran until the filtrate was colorless, and dried at 100 °C under vacuum overnight.

**Synthesis of Cu_3_-Ph-COF**

Cu_3_-Ph‑COF was assembled via solvothermal imine condensation. A mixture of Cu_3_-NH_2_ (0.02 mmol, 10.50 mg) and terephthalaldehyde (0.03 mmol, 4.00 mg) in o-dichlorobenzene/n-butanol/6 M acetic acid (5:5:1 v/v, 1.1 mL) was placed in a 10 mL Pyrex tube. The tube was degassed through three freeze-pump-thaw cycles and sealed under vacuum. It was then heated at 120 ℃ for 72 h. The resulting yellow crystalline solid was collected by filtration, washed repeatedly with anhydrous tetrahydrofuran until the filtrate was colorless, and dried at 100 °C under vacuum overnight.

**Section S3 Characterization of ligand and materials**

**
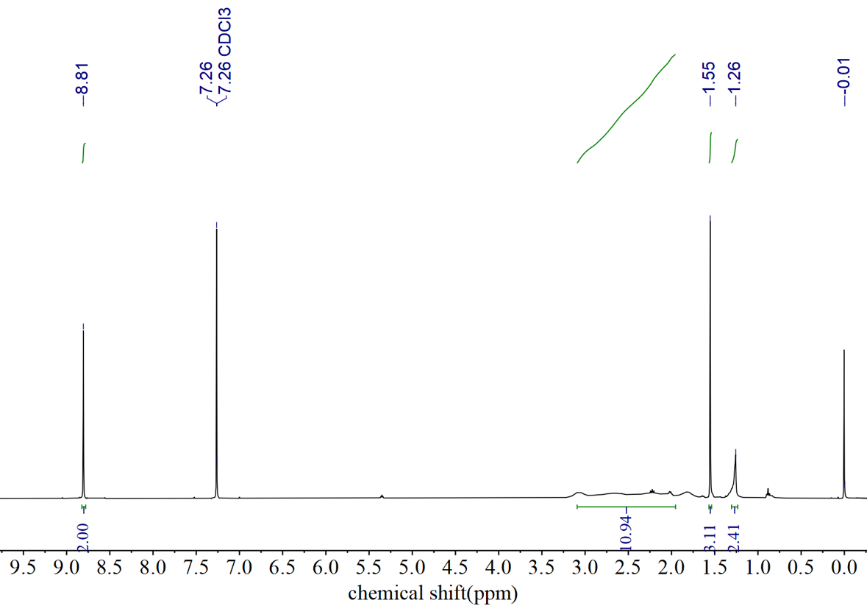
**

**Figure S1.** ^1^H NMR spectrum of CB-CHO.

**
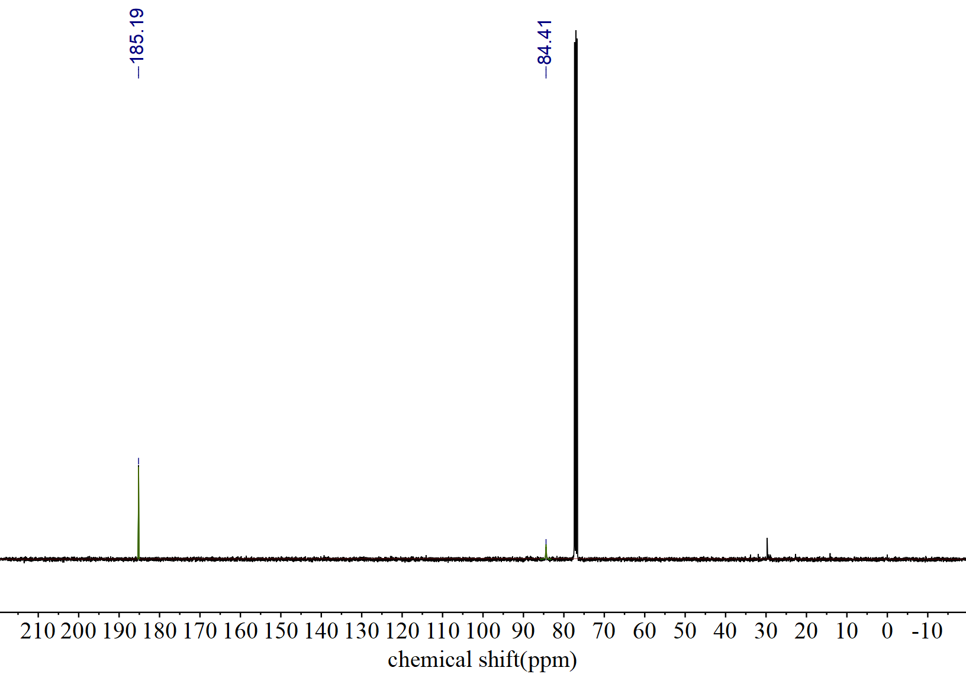
**

**Figure S2.** ^13^C NMR spectrum of CB-CHO.


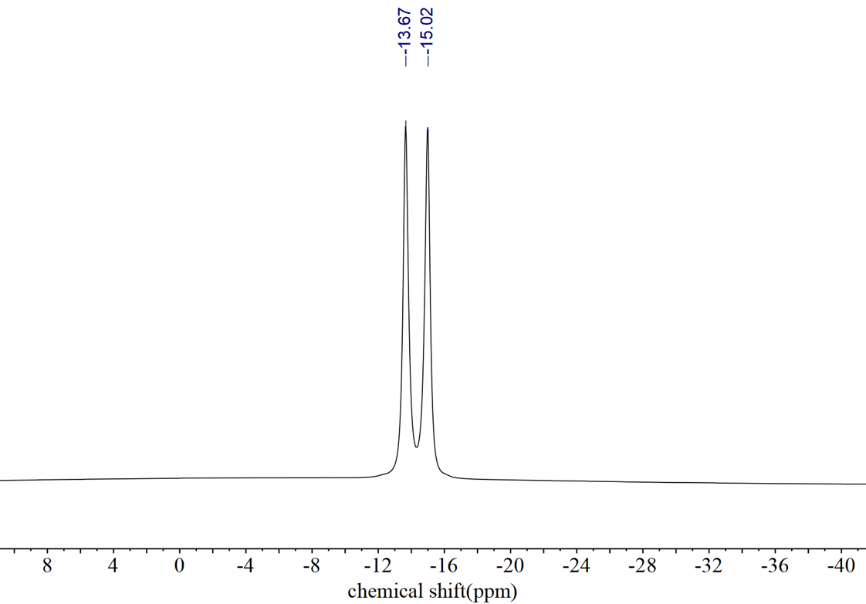


**Figure S3.** ^11^B NMR spectrum of CB-CHO.


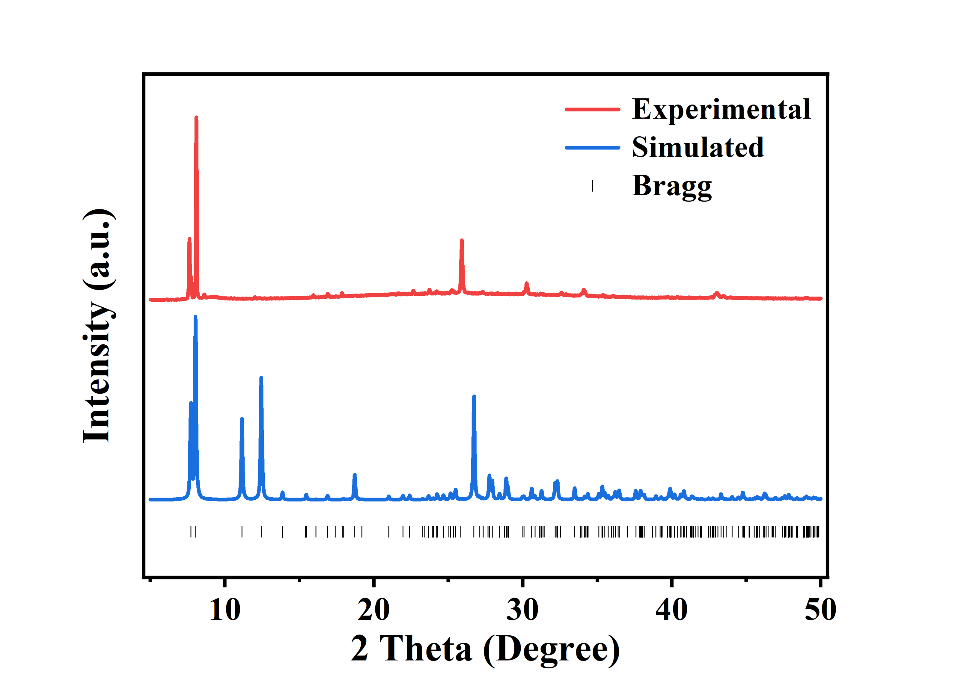


**Figure S4.** PXRD patterns of Cu_3_-NH_2_.


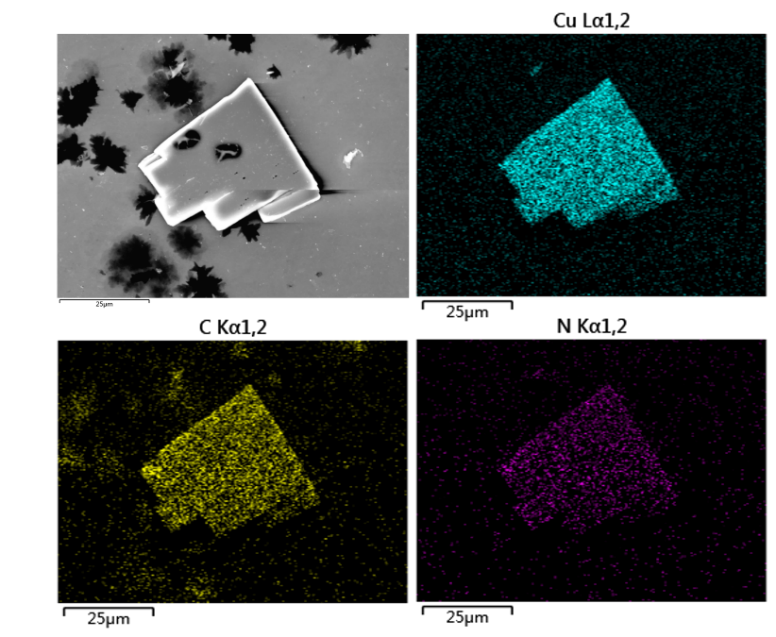


**Figure S5.** SEM image and the corresponding elemental mapping images of Cu_3_-NH_2_.

**
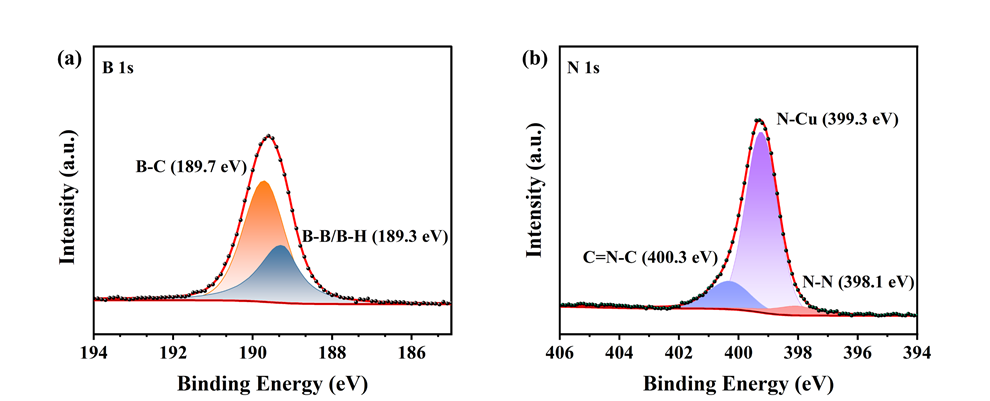
**

**Figure S6.** XPS spectrum of Cu_3_-CB-COF. (a) B 1s, (b) N 1s.


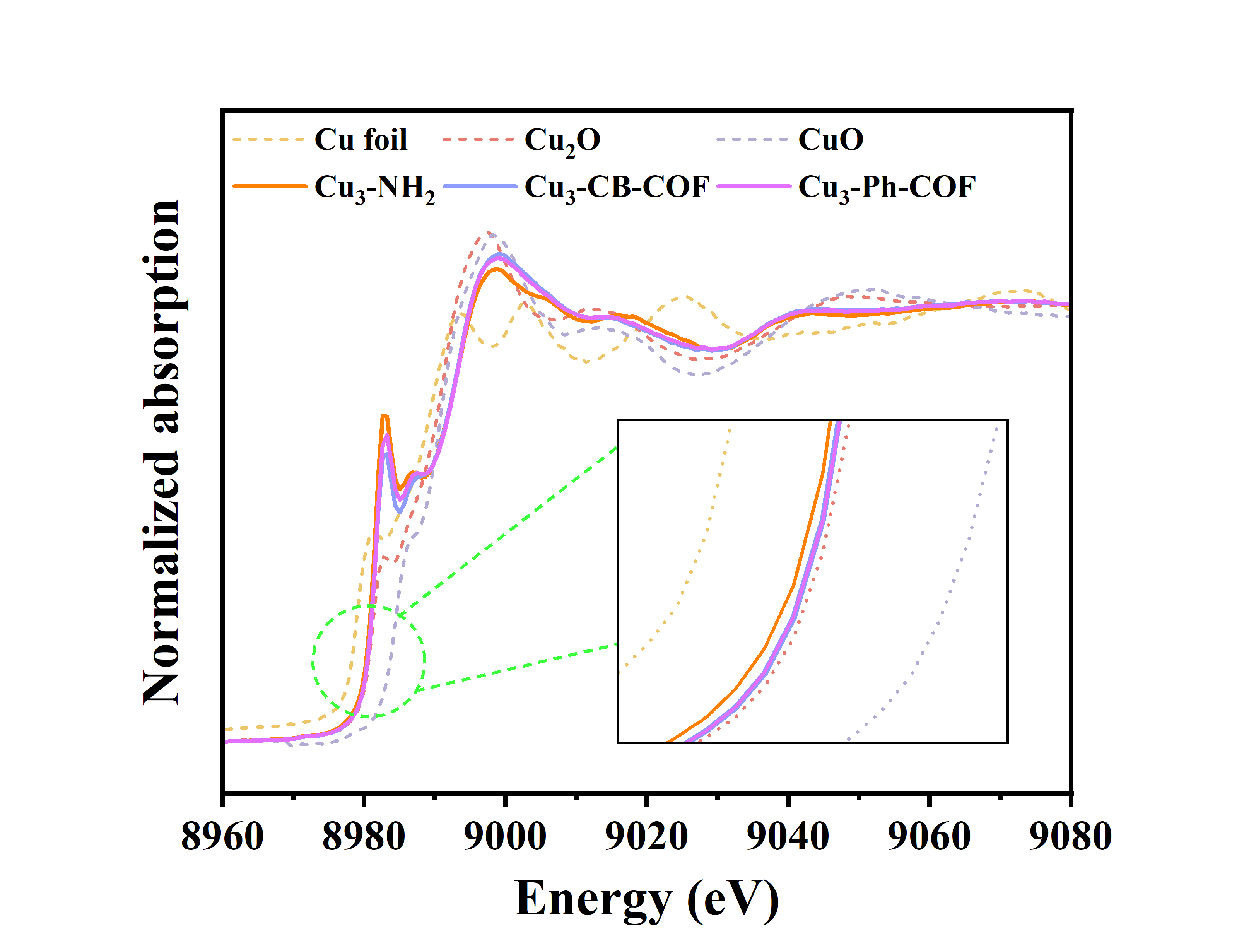


**Figure S7.** Normalized XANES spectra of the Cu K-edge for Cu_3_-NH_2_, Cu_3_-CB-COF and Cu_3_-Ph-COF.


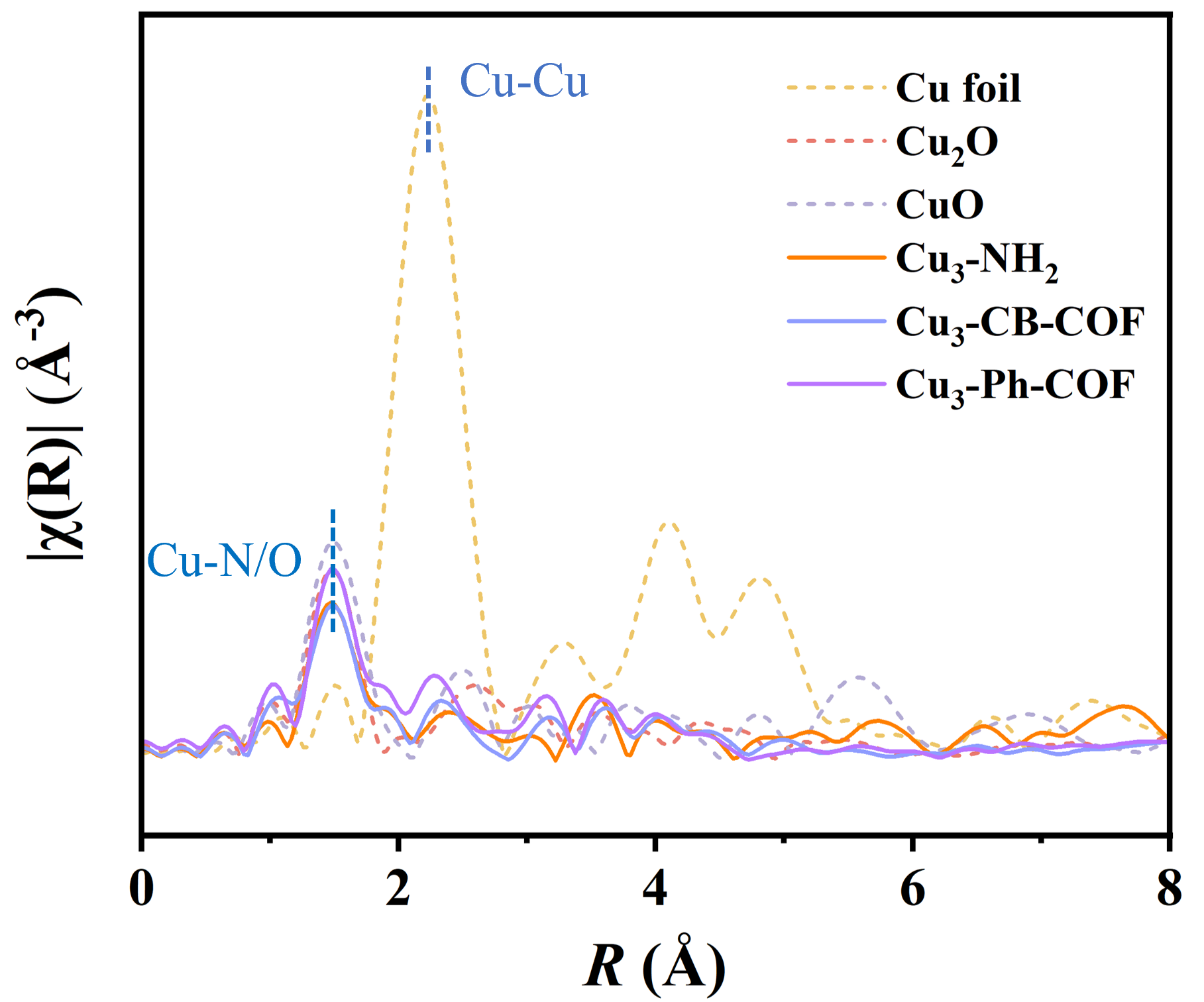


**Figure S8.** Fourier-transformed EXAFS spectra in the R-space of Cu_3_-NH_2_, Cu_3_-CB-COF and Cu_3_-Ph-COF.

**
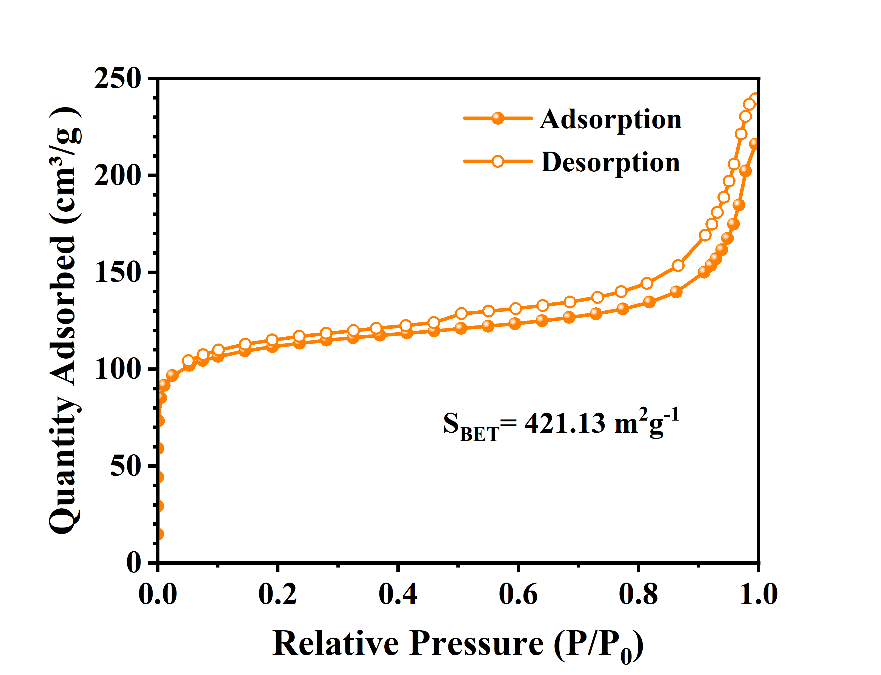
**

**Figure S9.** N_2_ adsorption-desorption isotherms of Cu_3_-CB-COF.


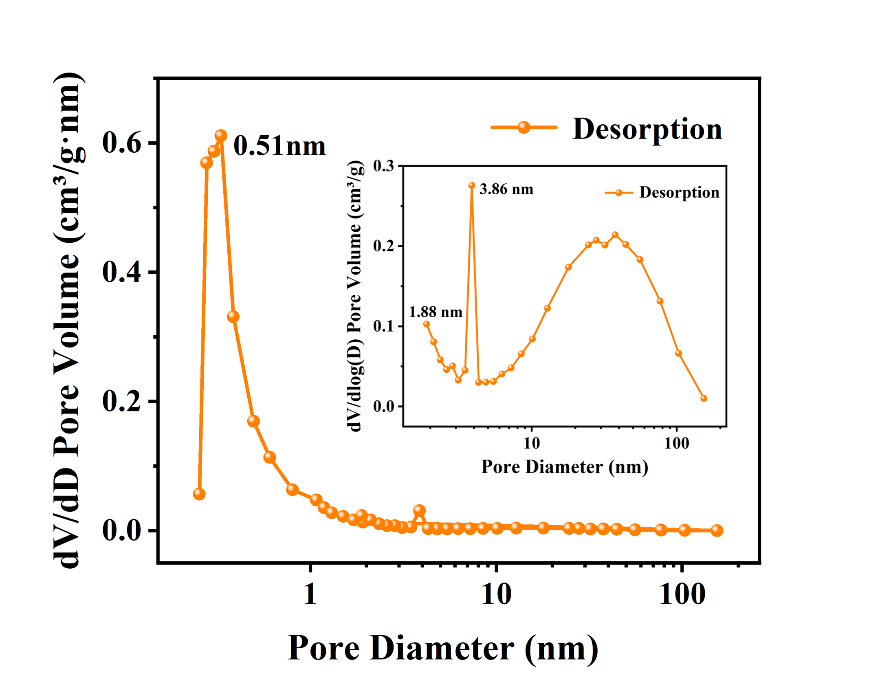


**Figure S10.** The pore size distribution profiles of Cu_3_-CB-COF.


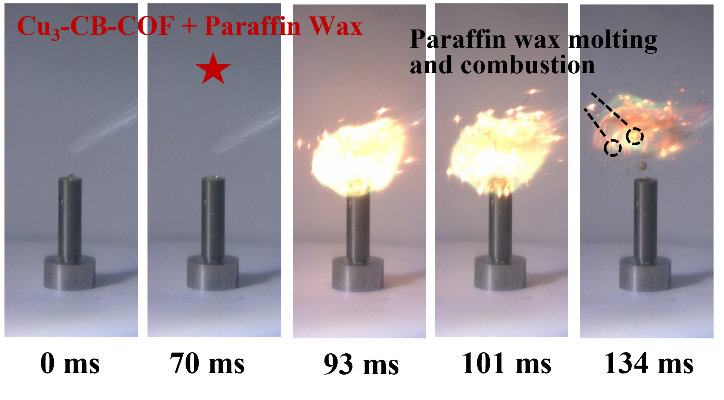


**Figure S11.** Hypergolicity drop test of Cu_3_-CB-COF and paraffin wax composite grain with HTP.


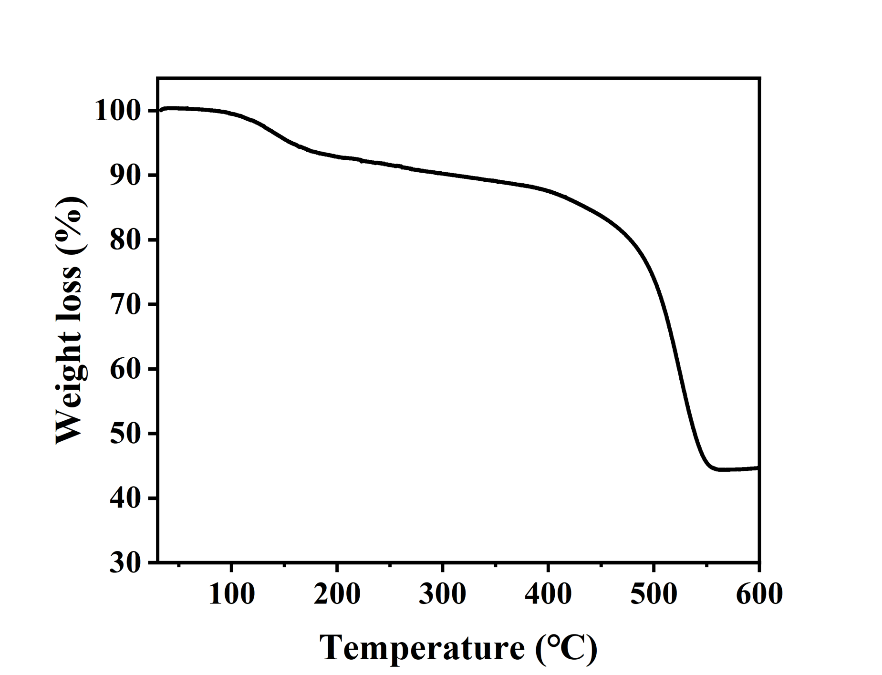


**Figure S12.** TGA curves of Cu_3_-CB-COF under N_2_ atmosphere.


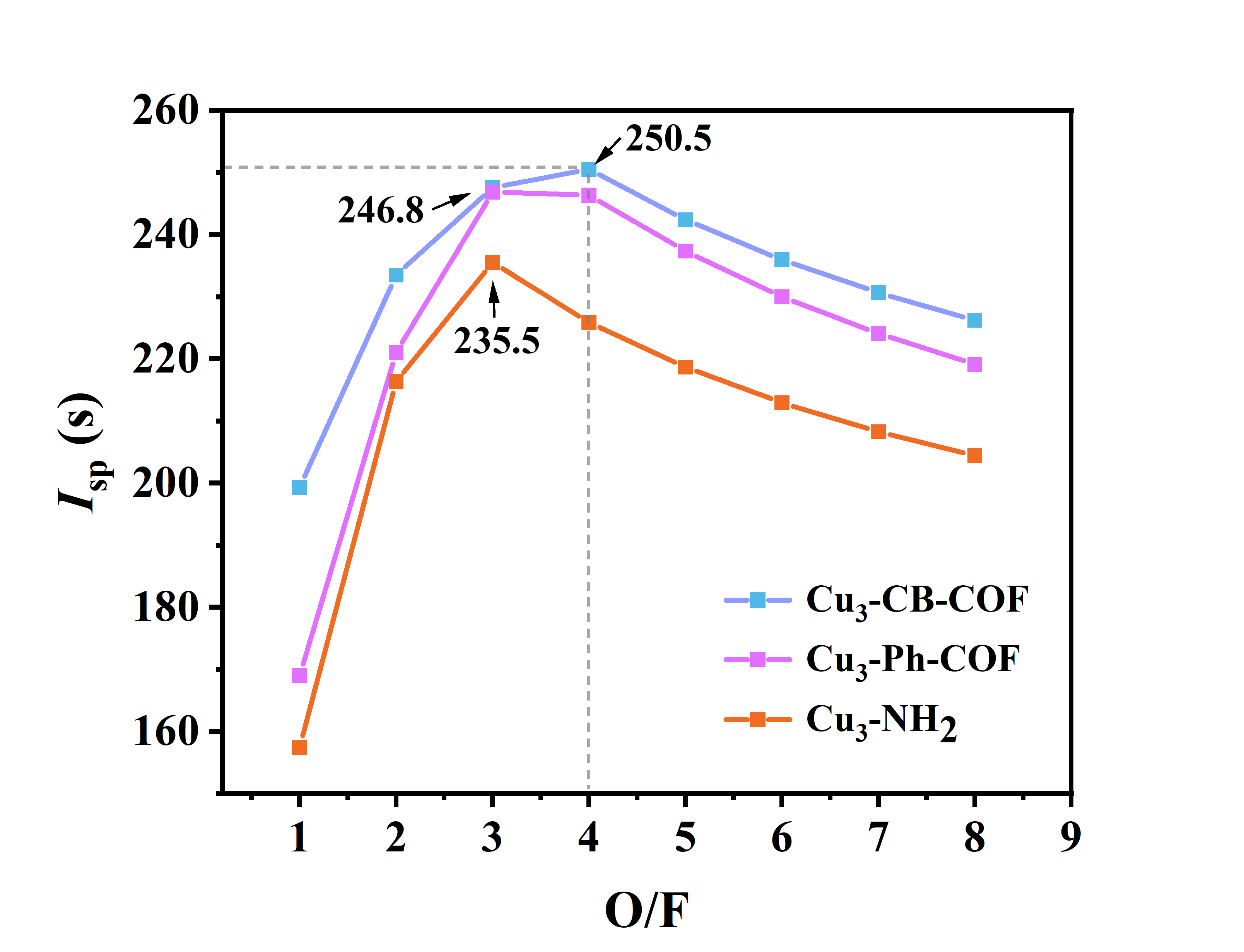


**Figure S13.** *I*_sp_ as a function of O/F for Cu_3_-CB-COF, Cu_3_-Ph-COF and Cu_3_-NH_2_.


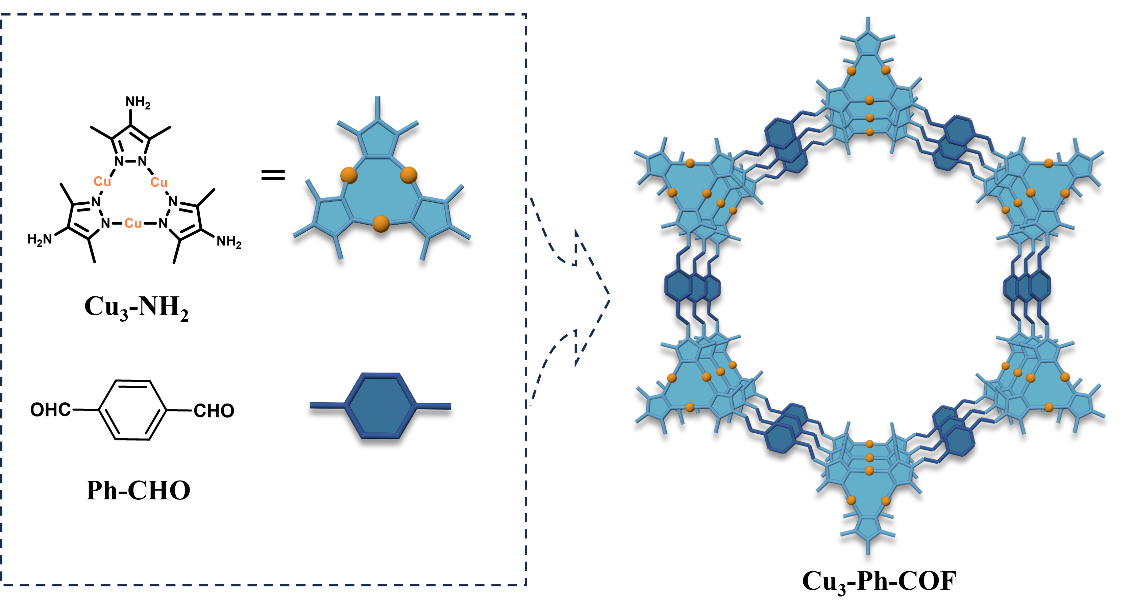


**Figure S14.** Schematic illustration of the synthesis of Cu_3_-Ph-COF.


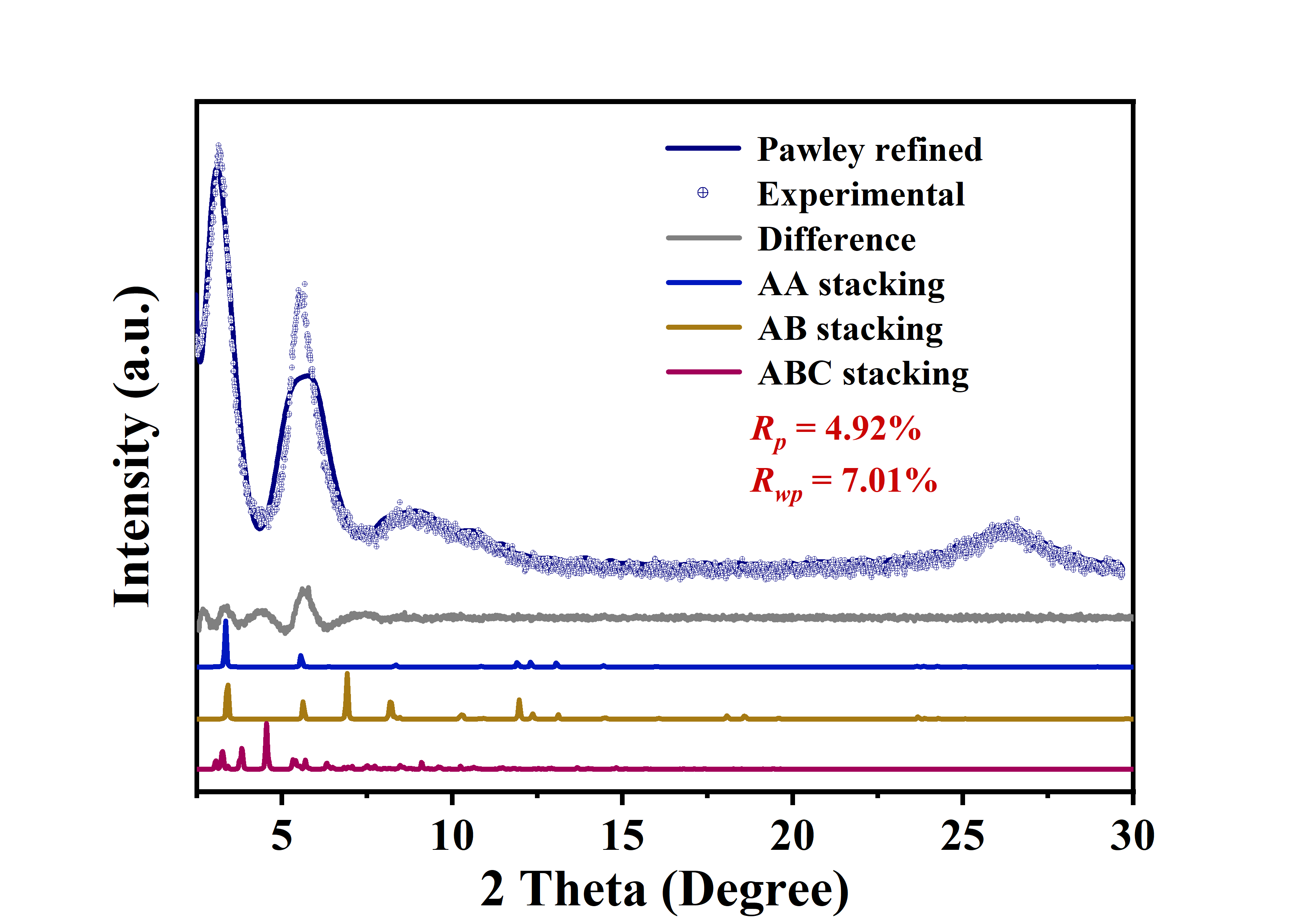


**Figure S15.** PXRD patterns of Cu_3_-Ph-COF.


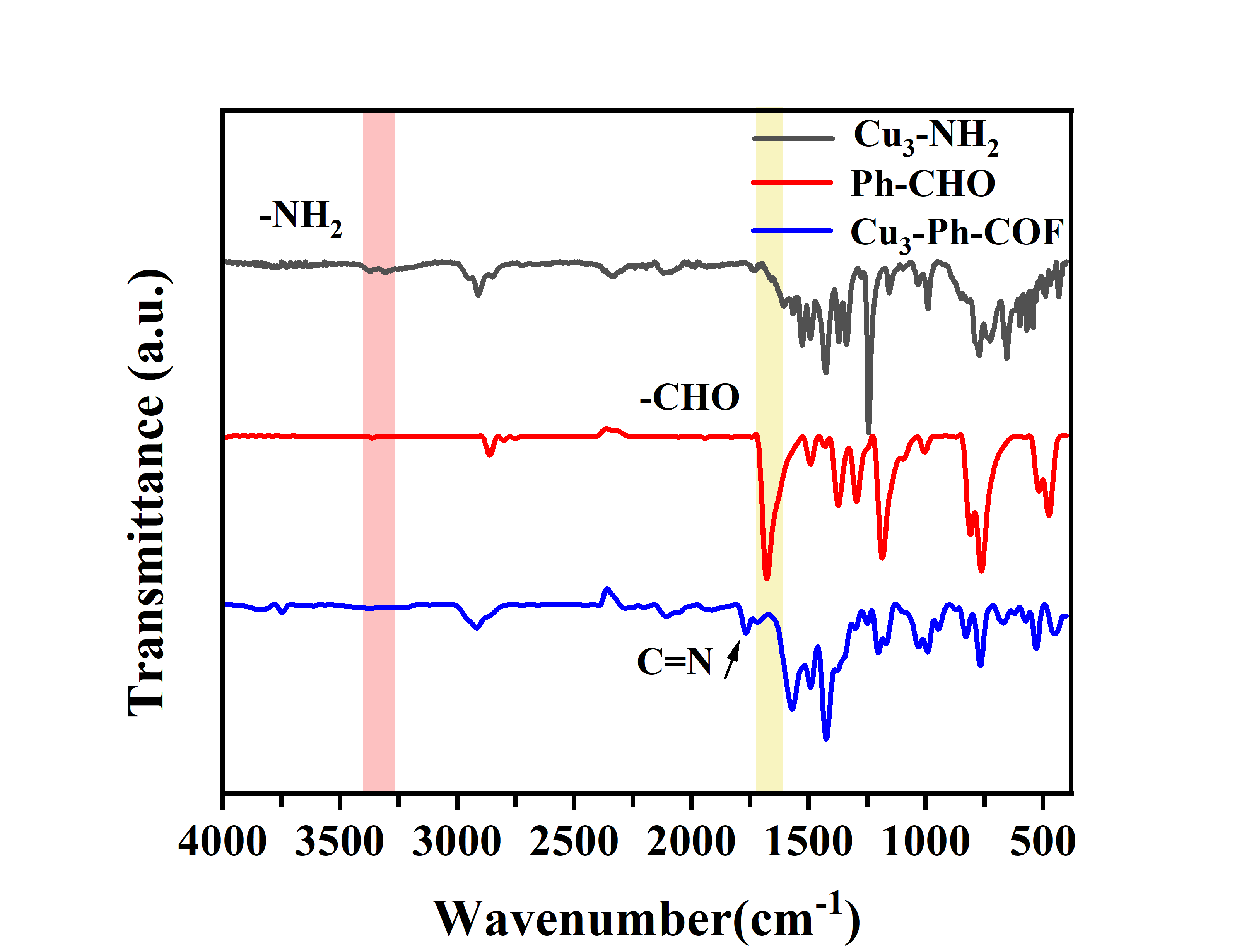


**Figure S16.** FT-IR spectra of Cu_3_-NH_2_, Ph-CHO and Cu_3_-Ph-COF.


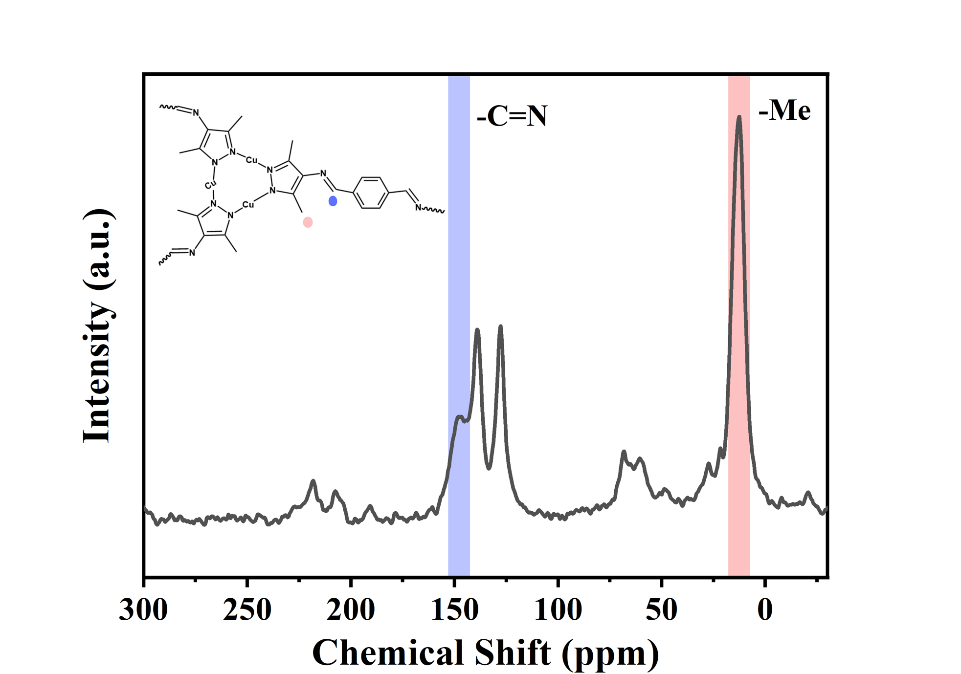


**Figure S17.** ^13^C CP/MAS NMR spectrum of Cu_3_-Ph-COF.


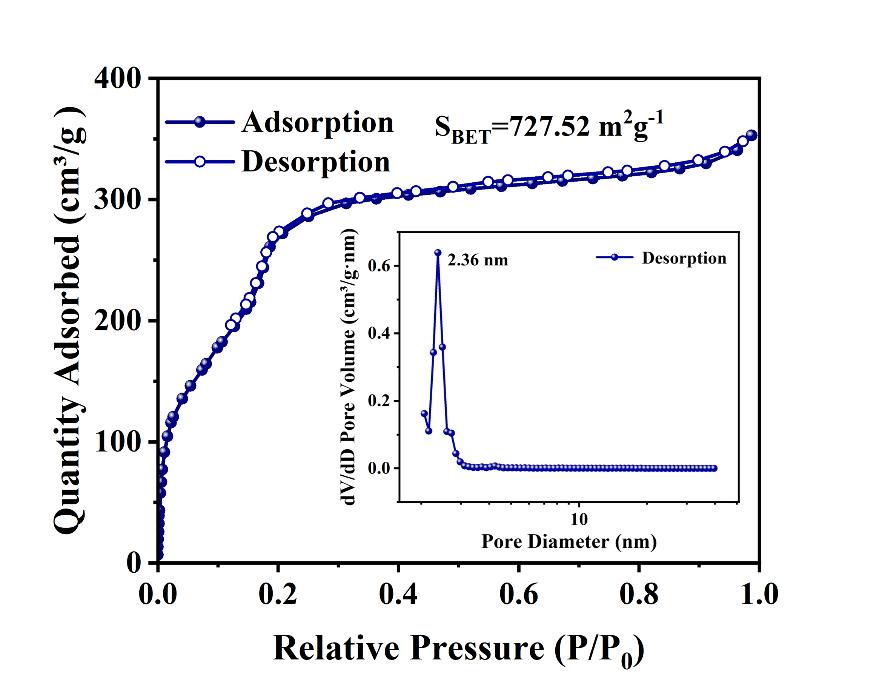


**Figure S18.** N_2_ adsorption-desorption isotherms and the pore size distribution profiles of Cu_3_-Ph-COF.


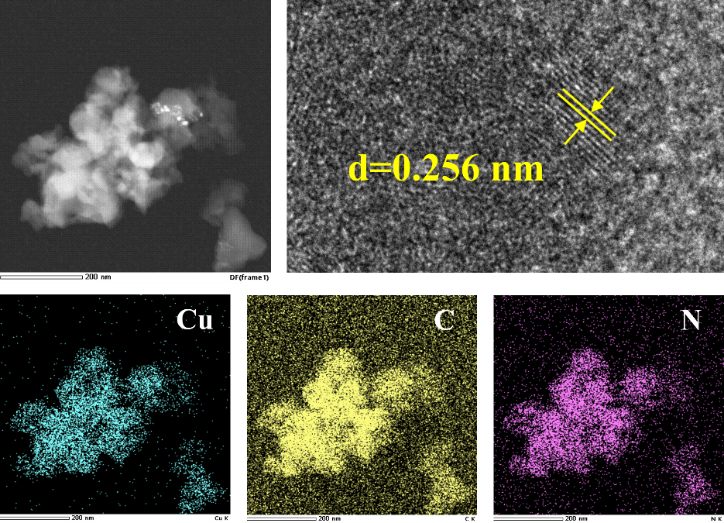


**Figure S19.** TEM and elemental mapping images of Cu_3_-Ph-COF.


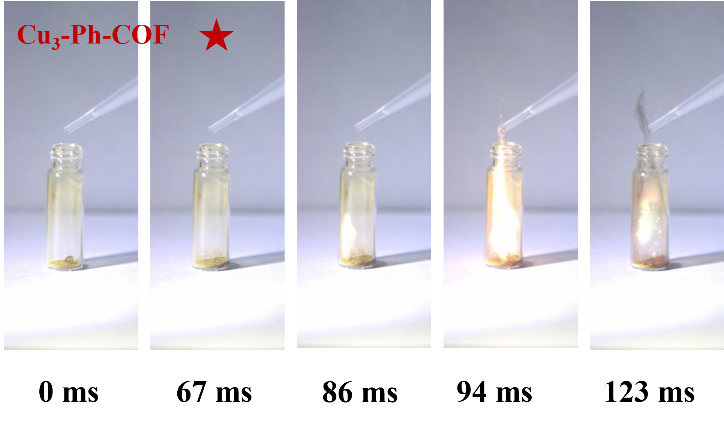


**Figure S20.** Hypergolicity drop tests of Cu_3_-Ph-COF with HTP.


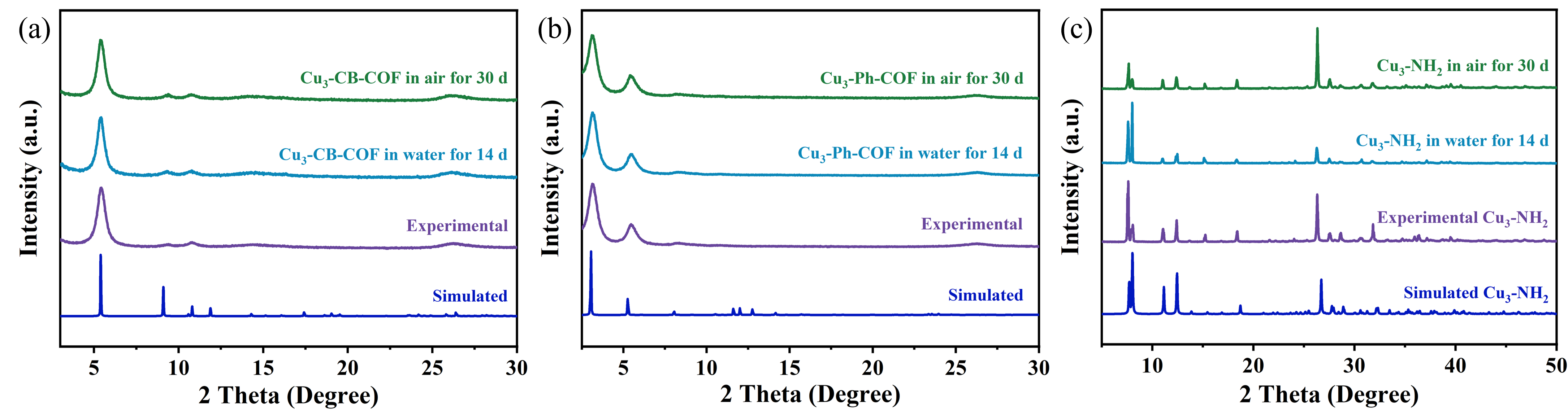


**Figure S21.** PXRD patterns of (a) Cu_3_-CB-COF, (b) Cu_3_-Ph-COF and (c) Cu_3_-NH_2_ for water and air stability at room temperature.


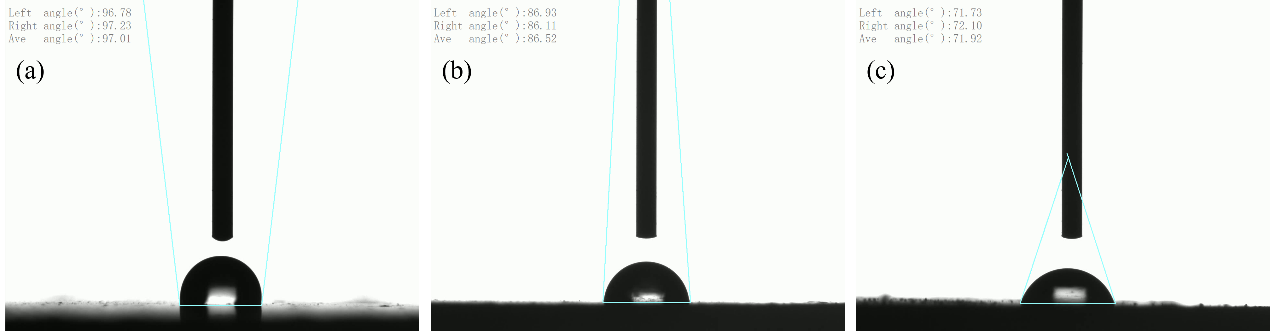


**Figure S22.** Contact angles of (a) Cu_3_-CB-COF, (b) Cu_3_-Ph-COF and Cu_3_-NH_2_ with pure water.

**Section S4 Theoretical Calculations**

The initial models of these systems with adsorbed molecules were constructed using Materials Studio (MS) software. All calculations were performed by using the density-functional theory (DFT) based Vienna ab initio simulation package (VASP).^5-8^ The generalized gradient approximation (GGA) in the form of the PBE exchange-correlation potential was adopted.^9,10^ DFT-D3 method of Grimme was considered to better describe the van der Waals interaction.^11^ The electronic wave functions were expanded in a plane-wave basis with a cutoff energy of 420 eV, and only the Γ-point was used for Brillouin zone sampling to prioritize computational efficiency for the large supercell. Geometric structure optimization was performed to simultaneously optimize atomic positions using the conjugate gradient algorithm, with convergence achieved when the energy differences fell below 1×10^-5^ eV per atom, residual atomic forces were reduced to 0.02 eV/Å. The minimum energy paths (transition states, TS) were conducted using climbing nudged elastic band (CI-NEB) method,^12^ and 3 images were inserted in between the IS and FS.

Partial charges of the molecules were evaluated using Bader charge analysis based on DFT-calculated electronic density.^13^ The charge density difference was evaluated by using the following equation:

$$\Delta\rho=\rho_{tot}-\rho_{substrate}-\rho_{adsorbate}$$

Where ρ_tot_ is the total charge density of the whole system, and ρ_substrate_ and ρ_adsorbate_ are the charge density of the materials and the H_2_O_2_, respectively. The quantitative charge transfer between Cu_3_-NH_2_, Cu_3_-CB-COF and H_2_O_2_ was calculated using the Bader method, employing Bader’s atom in molecule method to partition the total electron density into non-overlapping atomic volumes.


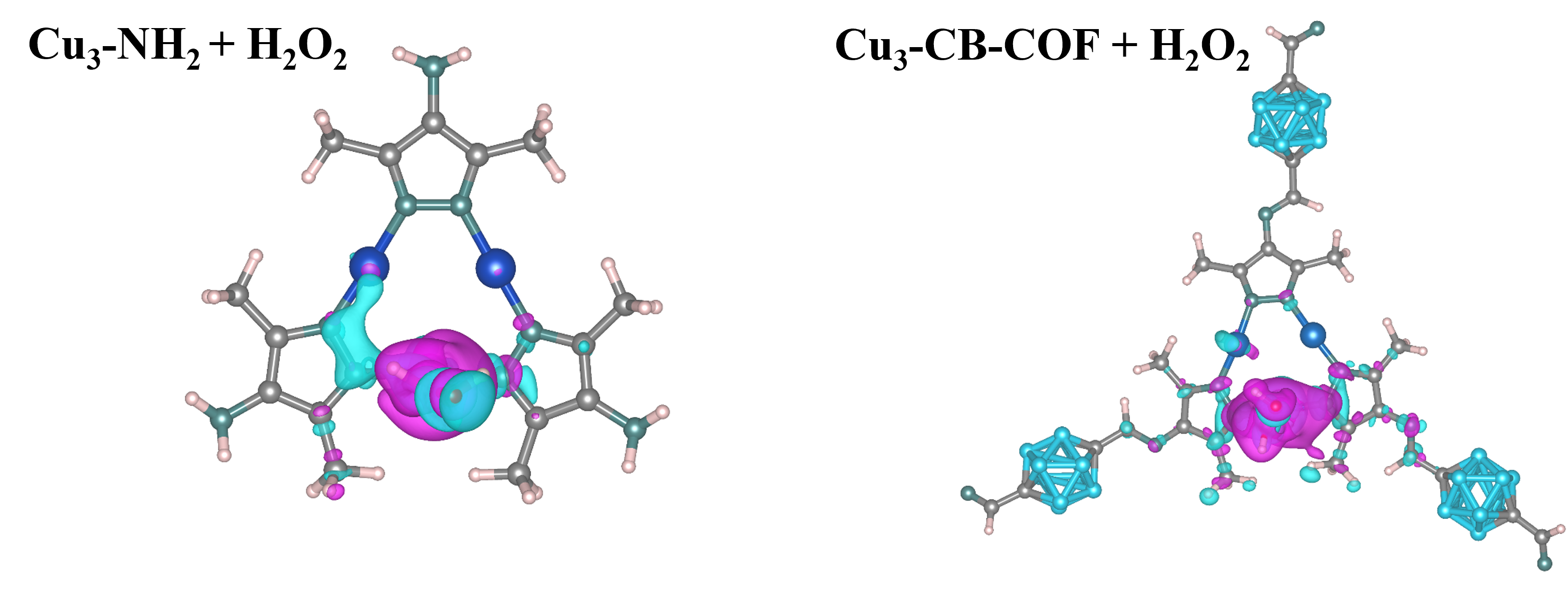


**Figure S23.** Differential charge density maps of Cu_3_-NH_2_ + H_2_O_2_ and Cu_3_-CB-COF + H_2_O_2_.


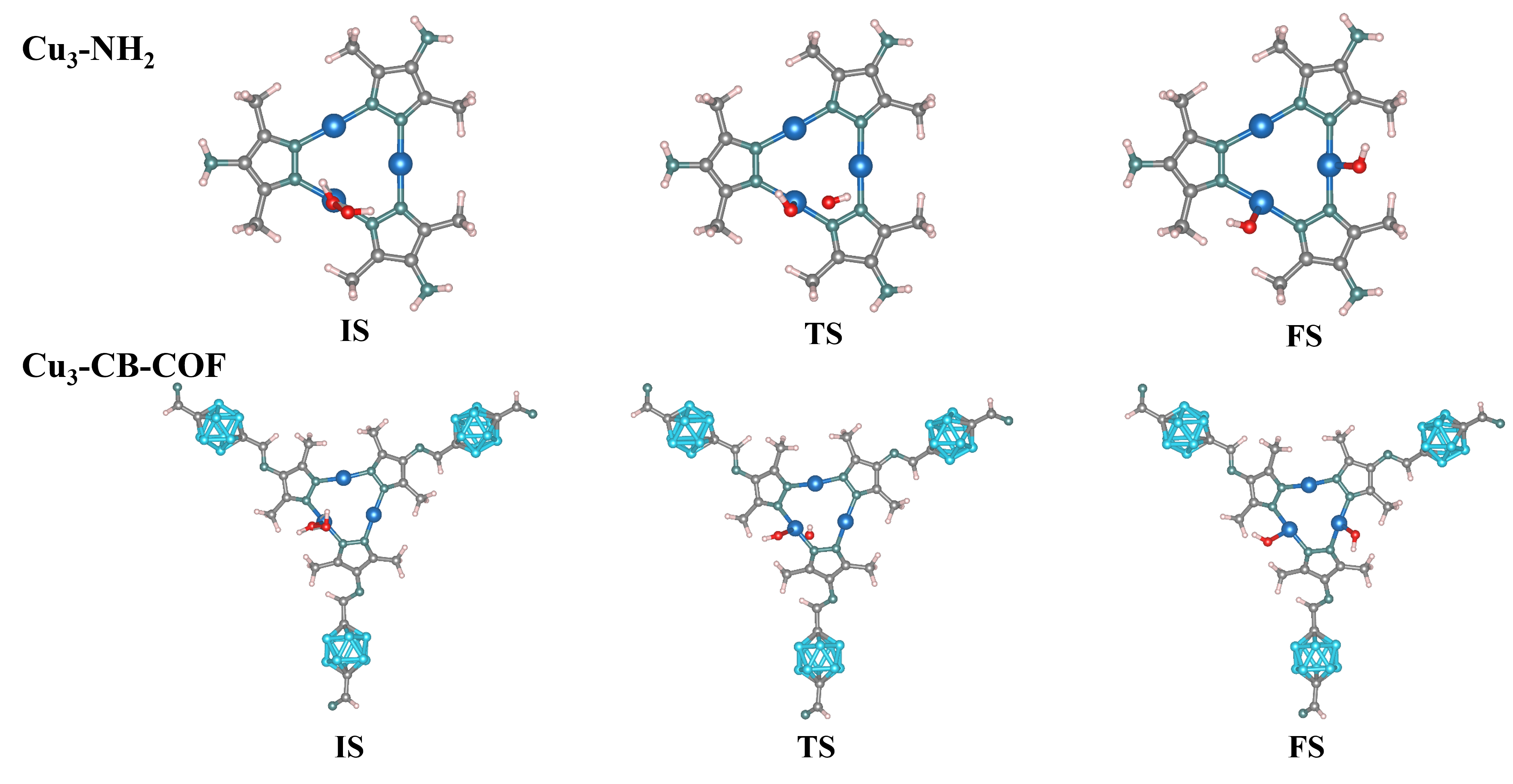


**Figure S24.** The calculated transition states of Cu_3_-NH_2_ and Cu_3_-CB-COF reacting with H_2_O_2_.


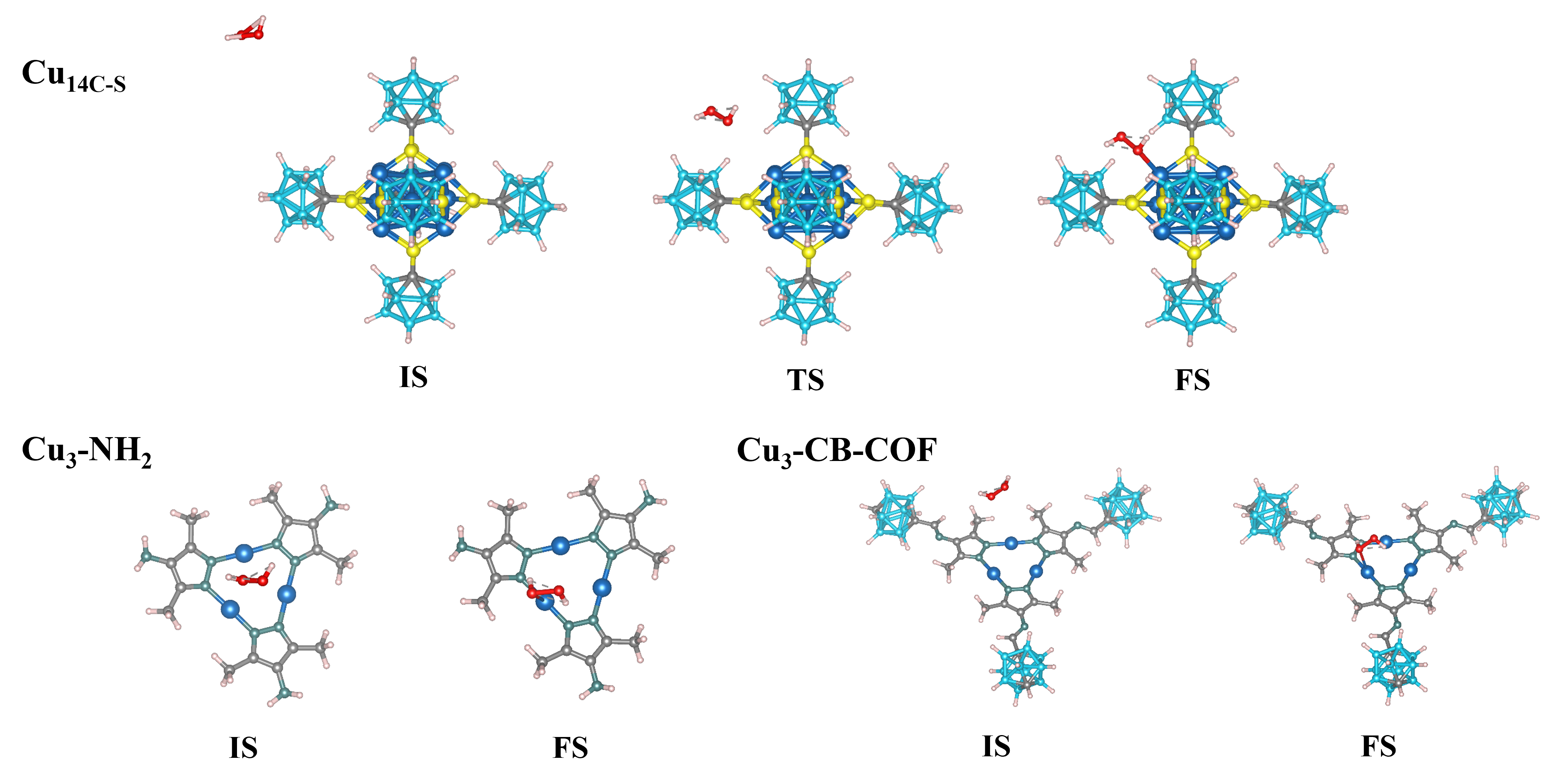


**Figure S25.** Diffusion path for H_2_O_2_ diffusing to hypergolic materials.

**References**

1 N. A. Mayer, D. M. Cupid, R. Adam, A. Reif, D. Rafaja, and H. J. Seifert, “Standard enthalpy of reaction for the reduction of Co_3_O_4_ to CoO,” *Thermochimica Acta* 652 (2017): 109–118, <https://doi.org/10.1016/j.tca.2017.03.011>.

2 B. J. McBride, M. J. Zehe, and S. Gordon, “NASA Glenn Coefficients for Calculating Thermodynamic Properties of Individual Species,” *NASA Technical Publication* TP-2002-211556 (2002), <https://ntrs.nasa.gov/citations/20020085330>.

3 X. Chen, J. Song, J. Zheng, et al., “Metal variance in multivariate metal-organic frameworks for boosting catalytic conversion of CO_2_,” *Journal of the American Chemical Society* 146 (2024): 19271–19278, <https://doi.org/10.1021/jacs.4c04556>.

4 X. Xu, Q. Cui, H. Chen, and N. Huang, “Carborane-based three-dimensional covalent organic frameworks,” *Journal of the American Chemical Society* 145 (2023): 24202–24209, <https://doi.org/10.1021/jacs.3c08541>.

5 G. Kresse and J. Hafner, “Ab initio molecular dynamics for liquid metals,” *Physical Review B: Condensed Matter and Materials Physics* 47 (1993): 558–561, <https://doi.org/10.1103/PhysRevB.47.558>.

6 G. Kresse and J. Hafner, “Ab initio molecular dynamics for open-shell transition metals,” *Physical Review B: Condensed Matter and Materials Physics* 48 (1993): 13115–13118, <https://doi.org/10.1103/PhysRevB.48.13115>.

7 G. Kresse and D. Joubert, “From ultrasoft pseudopotentials to the projector augmented-wave method,” *Physical Review B: Condensed Matter and Materials Physics* 59 (1999): 1758–1775, <https://doi.org/10.1103/PhysRevB.59.1758>.

8 G. Kresse and J. Furthmüller, “Efficiency of ab-initio total energy calculations for metals and semiconductors using a plane-wave basis set,” *Computational Materials Science* 6 (1996): 15–50, <https://doi.org/10.1016/0927-0256(96)00008-0>.

9 J. P. Perdew, K. Burke, and M. Ernzerhof, “Generalized gradient approximation made simple,” *Physical Review Letters* 77 (1996): 3865–3868, <https://doi.org/10.1103/PhysRevLett.77.3865>.

10 P. E. Blöchl, “Projector augmented-wave method,” *Physical Review B* 50 (1994): 17953–17979, <https://doi.org/10.1103/PhysRevB.50.17953>.

11 X. Wu, M. C. Vargas, S. Nayak, V. Lotrich, and G. Scoles, “Towards extending the applicability of density functional theory to weakly bound systems,” *The Journal of Chemical Physics* 115 (2001): 8748–8757, <https://doi.org/10.1063/1.1412004>.

12 G. Henkelman, B. P. Uberuaga, and H. Jónsson, “A climbing image nudged elastic band method for finding saddle points and minimum energy paths,” *The Journal of Chemical Physics* 113 (2000): 9901–9904, <https://doi.org/10.1063/1.1329672>.

13 R. F. W. Bader, “A quantum theory of molecular structure and its applications,” *Chemical Reviews* 91 (1991): 893–928, <https://doi.org/10.1021/cr00005a013>.
